# Correction: Biocontrol of rice blast by Pseudomonas mosselii PR5 through seed priming and foliar application reduces reliance on chemical pesticides

**DOI:** 10.1371/journal.pone.0357646

**Published:** 2026-09-03

**Authors:** The PLOS One Staff

## Notice of Republication

This article was republished on August 11, 2026, to correct errors with author affiliations introduced during the typesetting process. The publisher apologizes for the errors. Please download this article again to view the correct version. The originally published, uncorrected article and the republished, corrected articles are provided here for reference.

## Supporting information

S1 File Originally published, uncorrected article. (PDF)

S2 File Republished, corrected article. (PDF)
