# Supplementary material for: Biocontrol of rice blast by Pseudomonas mosselii PR5 through seed priming and foliar application reduces reliance on chemical pesticides
Source: PLoS One. 2026 Jun 18;21(6):e0351650. doi: 10.1371/journal.pone.0351650 (PMC13278436; doi:10.1371/journal.pone.0351650)
Supplement: Attachment — Submitted filename: Response to reviewers comments PLOS ONE.docx [file pone.0351650.s002.docx]

**Author Response to** **Reviewers' comments**

**Reviewer#1:**

**Reviewers' comments 1:** This study addresses a relevant and important topic: the search for sustainable alternatives to chemical fungicides for controlling rice blast, a major threat to global food security. The experimental design is comprehensive, testing multiple application methods of the biocontrol agent Pseudomonas mosselii PR5 across three susceptible rice genotypes. The results demonstrate clear potential for PR5, particularly the combination treatments (SP+BCF and SeP+BCF), in reducing disease severity and promoting plant growth and yield. The manuscript is generally well-structured, but several critical flaws in methodology, data presentation, and interpretation must be addressed before it can be considered for publication.

**Response1:** Thank you very much for your valuable comments and important suggestion. We believe your comments and suggestions would significantly improve our manuscript. We have addressed all the comments point-by-point below.

**Reviewers' comments 2:** The phrase "including an absolute control, a pathogen-inoculated control, a fungicide control, and five PR5 application modes" is not clear. It is not clear which control group receives the pathogen and which does not.

**Response 2:** The absolute control did not receive the blast pathogen. This treatment also did not receive chemical fungicide or PR5 as a biocontrol agent.

All other treatments received blast pathogen. The negative control received the blast pathogen only. In addition to blast pathogen, positive control received chemical fungicide; and the PR5 treatment group received PR5 trough seed priming (SP), seedling priming (SeP), bacterial culture filtrate (BCF); SP+ BCF and SeP + BCF.

To remove the confusion, we have presented the treatments in a tabular form in the methodology section in the revised manuscript as follows:

| Name | Pathogen inoculation | Treatment applied |
| --- | --- | --- |
| Absolute control or Untreated control (AC) | No | No |
| Negative control or inoculated control (NC) | Yes | No |
| Positive control (PC) | Yes | Yes, fungicide as known effective treatment |
| Seed priming (SP) | Yes | Yes, PR5 as seed priming |
| Seedling priming (SeP) | Yes | Yes, PR5 as seedling priming |
| Bacterial culture filtrate (BCF) | Yes | Yes, PR5 culture filtrate as foliar application |
| SP + BCF | Yes | Yes, combined application of PR5 as seed priming and BCF foliar application |
| SeP + BCF | Yes | Yes, combined application of PR5 as seedling priming and BCF foliar application |

**Reviewers' comments 3:** The term "seedling priming" (SeP) is introduced but is not a standard term in the literature. "Seed priming" is common

**Response 3:** We use the term “**seed priming**” when we **primed the seeds** of rice with bacterial solution and designated as “**SP**” in short form. However, when we **primed the seedlings** then the method is termed as “**seedling priming**” and designated as “**SeP**”. Rice seed is used for direct seeding and seedling is used for transplanted cultivation. Therefore, two distinct priming terms viz. ‘seed priming’ and ‘seedling priming’ were used. Often in scientific paper these two terms used equivalently and term as “seed priming” but we used separate term as these two methods were used as two separate treatments in our experiment.

**Reviewers' comments 4:** Bacterial culture filtrate (BCF)-Does it refer to a spray of the liquid growth medium containing bacterial metabolites but no live cells? Or is it a suspension of live bacteria?

**Response 4:** In the current experiment bacterial culture filtrate (BCF) refers the supernatant of bacterial liquid culture. Upon centrifuging the liquid culture of bacteria, the supernatant was used as bacterial culture filtrate. It is not suspension of live bacteria.

**Reviewers' comments 5:** The statement "SP+BCF achieved the highest yield and outperformed the fungicide in disease suppression" is main conclusion. However, the abstract does not provide the actual disease severity data (e.g., "SP+BCF reduced disease severity by X% compared to the pathogen control, compared to Y% for the fungicide").
For example, "SP+BCF produced the lowest AUPDC in V1 and V3" – how much lower? And crucially, how did it compare to the fungicide in those varieties? Was the difference statistically significant?

**Response 5:** Few numerical data on disease suppression is included to make the statement clearer in the revised manuscript as follows:

In V1 and V3, SP + BCF even outperformed the chemical fungicide in controlling the disease. For instance, at 28^th^ DAI, SP+ BCF reduced the disease severity by 41.46% and 40.57 % compared to negative control and 5.89% and 14.58 % compared to chemical fungicide treatments in V1 and V3, respectively.

Few other numerical data also added in the result section and the texts are marked.

**Reviewers' comments 6:** The final sentence of the results section ("SP+BCF achieved the highest yield and outperformed the fungicide in disease suppression") largely repeats the point made earlier about SP+BCF producing the lowest AUPDC and achieving the highest yield

**Response 6**: The final sentence of the result section actually expressed the superior performance of SP + BCF over chemical fungicide in yield and yield attributes. The earlier sentence in disease matrix part, was highlighted the superior performance of SP + BCF in disease control over all other treatments, especially over chemical fungicide.

However, to avoid the similarity in write up, one line in disease subsection in result is revised as follows:

In V1 and V3, SP + BCF even outperformed the chemical fungicide in controlling the disease. For instance, at 28^th^ DAI, SP+ BCF reduced the disease severity by 41.46% and 40.57 % compared to negative control and 5.89% and 14.58 % compared to chemical fungicide treatments in V1 and V3, respectively.

**Reviewers' comments 7:** The introduction should articulate the specific knowledge gap that this study aims to fill.

**Response 7:** Introduction is revised according to the suggestion.

**Reviewers' comments 8:** The introduction should set up the hypothesis that different application methods (priming vs. foliar) might have different effects, and that combining them could be synergistic. Currently, the introduction presents PR5 as a promising agent but doesn't foreshadow the central question of the paper: What is the best way to use it?

**Response 8:** The Introduction section is revised and the hypothesis that the bacterial efficacy could be different based on application method.

**Reviewers' comments 9:** Cite some recent studies in the introduction <https://doi.org/10.1016/j.bcab.2020.101729>, <https://doi.org/10.1002/aoc.5190>, <https://doi.org/10.1016/j.micpath.2024.107084>

**Response 9:** The mentioned papers are included as citation in Introduction section.

**Reviewers' comments 10:** The treatment list in lines 106-109 is confusing and contains an error. It states: "T0, absolute control; T1, negative control; T2, positive control (commercial fungicide foliar application); ... T6, seed priming + BCF foliar; and 76, seedling priming + BCF foliar application." Later, in lines 128-132, it mentions a fungicide sprayed in "treatments T1". This is a major inconsistency. Which one is the fungicide treatment? A "negative control" typically receives no treatment and no pathogen, while a "positive control" receives a known effective treatment (like a fungicide) and the pathogen. The authors must clarify the treatment codes and ensure they are used consistently throughout the manuscript, including in all tables and figures.

**Response10:** Thank you very much for your valuable suggestion.

In our case we referred the treatment that receive no pathogen and no other chemical or biological agent a “absolute control”. The negative control is referred to the treatment that received pathogen but no fungicide or biocontrol agent and the positive control referred to the treatment that received the pathogen and a known fungicide as you mentioned.

There was a Typo in line number 128 (T1 was written instead of T2) that we have corrected in the revised manuscript.

The whole manuscript including all the figures are checked and revised for the consistency of the data code as per the suggestion. The clarification of the treatment code also given with each figure caption.

To clear the treatment codes, we added a table in the methodology section the revised manuscript which is already added in the response 2.

**Reviewers' comments 11:** To properly compare the biocontrol agent to the chemical standard, there must be a treatment that receives the pathogen and is sprayed with the fungicide but does not receive any PR5 treatment. As described, it's impossible to separate the effect of the fungicide from the potential confounding effects of PR5 if the fungicide was applied to the same plants as PR5 treatments.

**Response11:** Thank you very much for your valuable suggestion. We do agree with you.

It is noted that we did not use chemical fungicide and PR5 in same treatment. No plants received both chemical fungicide and PR5 in any form of application. The only treatment that received chemical fungicide is the “positive control”. The other plants from T3-T7 received PR5 in different mode of applications. Treatments T0 and T1 did not receive chemical fungicide or PR5. There was a Typo in line number 128 (T1 was written instead of T2) that we have corrected now.

**12. Reviewers' comments 12:** The method describes the pathogen stock as a "paper disc" (Line 143). This is an unusual and non-standard method for long-term storage of M. oryzae. Typically, cultures are stored on agar slants, in sterile soil, or on filter paper in a dried state. The phrase "presented as a paper disc" is unclear.

**Response12:** We agree that the phrase “presented as a paper disc” was unclear. The pathogen isolate was not stored as an ordinary paper disc; rather, it was preserved as a dried sterile filter paper disc (round piece of dried sterile filter paper) colonized by *Magnaporthe oryzae*. This is a recognized method for maintaining M. oryzae isolates for long-term storage.

The sentence is revised to make it clearer in the revised manuscript as follow:

The stock culture of *Magnaporthe oryzae* was preserved as a dried sterile filter paper disc colonized with the fungus and was revived on potato sucrose agar medium before inoculum preparation.

**Reviewers' comments 13:** Figure 1-3 and figure 9, the figures quality is poor these must be readable.

Response13: Figures 1–3 and Figure 9 have been reconstructed to improve image quality, and all other figures were carefully checked and revised for consistent figure captions.

Please be noted that, the experimental design involved a complex setup comprising three rice genotypes and eight treatments. Since the data were analyzed using two-way ANOVA, two sets of lettering were required to indicate statistical significance, which made the figures appear crowded and reduced the possible font size. Nevertheless, we adjusted the lettering and formatting to the maximum readable size that could be accommodated within each figure while maintaining clarity and completeness of the statistical information. Orientation of the figures are also changed and rearranged to increase the clarity.

**Reviewer #2:**

**1. Reviewers' comments 1:** The manuscript investigates the biocontrol efficacy of the endophytic bacterium Pseudomonas mosselii PR5 against rice blast (Magnaporthe oryzae) across three susceptible rice genotypes. The research assesses different application techniques—such as seed priming, seedling priming, and bacterial culture filtrate (BCF) foliar spray—and contrasts their efficacy with that of a commercial fungicide. The findings demonstrate that combined applications, especially SP+BCF and SeP+BCF, significantly mitigate disease severity (PDI and AUDPC) while concurrently promoting plant growth and yield metrics, often surpassing the efficacy of chemical fungicides.
The study addresses an important issue in sustainable agriculture and plant pathology, fitting perfectly within the broad scope of PLOS ONE. The dual-action assessment of PR5 (growth promotion and disease suppression) is commendable. However, I have identified several critical methodological gaps, instances of confusing terminology, and a significant overreach in the Discussion section, in which prior findings are conflated with current data. Before considering the manuscript for publication, the authors should address these issues.

**Response 1:** Thank you very much for your constructive comments and suggestions that will be helpful to improve our manuscript. We replied to each comments point-by-point below and corrections has made in the manuscript as and where necessary.

**Reviewers' comments 2:** Section 2.2 details the pot preparation but completely omits the statistical design of the experiment. Was this a Completely Randomized Design (CRD) or a Randomized Complete Block Design (RCBD)?
**Response 2:** Thank you for your valuable suggestion.

The experimental design was Completely Randomized Design (CRD). We included the statistical design in the corrected manuscript in Section 2.2.

**Reviewers' comments 3:** Furthermore, the authors note in the Discussion that pots were kept 25 cm apart in natural field conditions and that T0 (absolute control) pots were "physically separated by polyethylene sheets." This crucial methodological information should be included in Section 2.2, rather than being presented as an afterthought in the Discussion to clarify anomalous data. Please detail the exact spatial arrangement, post-inoculation environmental conditions, and randomization protocol.

**Response 3:** Actually, the pots that received the blast pathogen was physically separated when blast pathogen was inoculated. As all other treatments except absolute control received blast pathogen, so all the pots except T0 treatment, were taken into a controlled chamber that was separated and isolated. However, after successful disease development the pots were again kept into the same place as they were before.

The spatial arrangement, post-inoculation environmental conditions, and randomization protocol is included in the revised manuscript.

**Reviewers' comments 4:** In the Discussion, the authors state that PR5 produces IAA, solubilizes phosphorus, and produces siderophores and HCN. None of these biochemical assays were conducted or presented in the current manuscript. While these may be established traits of PR5 from the authors' previous works, the current phrasing makes it appear as though these mechanisms were proven in this specific study. The authors must strictly separate their current phenotypic/disease findings from previous in vitro characterizations. Please revise these sections.

**Response 4:** The mentioned section (marked as red) in the Discussion is revised to remove any confusion about the finding of current study and the findings of our previous study about the isolate as follows:

The mechanism of growth improvement by PR5 was reported in earlier studies, where this bacterium was found capable of producing significant amounts of indole-3 acetic acid (IAA), fixing atmospheric nitrogen, solubilize insoluble phosphorus, Fe, zinc, and silicon [36-37].

**Reviewers' comments 5:** In Lines 132-135, the authors mention centrifuging the bacterial solution (OD600 = 1) and mixing the supernatant with CMC for foliar application. Later, in line 225, the solution is referred to as a "bacterial cell-free (BCF)" application. Was this supernatant passed through a microbiological filter (e.g., 0.22 µm) to guarantee it was entirely cell-free? If no filtration was performed, viable cells likely remained in the supernatant, making the term "cell-free" scientifically inaccurate. Please clarify the exact sterilization/filtration protocol. If it was not filtered, the terminology must be corrected throughout the manuscript.

**Response 5:** We actually used the term bacterial culture filtrate (BCF) in the treatment. The bacterial liquid culture solution was centrifuged and the supernatant was filtered through 0.22 µm syringe filter and used as BCF for foliar application by mixing with CMC.

However, the line 225 (L- 249 in the revised manuscript) is corrected as “bacterial culture filtrate (BCF)” to remove any inconsistency in writing or confusion.

**Reviewers' comments 6:** In Lines 124-125, T1 (pathogen only) is labeled the "negative control" and T2 (fungicide) is labeled the "positive control." In plant pathology, a pathogen-only treatment is typically considered a positive control for disease development. To avoid reader confusion, I recommend utilizing descriptive nomenclature throughout the manuscript.
**Response 6:** In our experiment the treatment group is labelled as absolute control/untreated control, negative control that received pathogen but no other treatment and positive control that received pathogen and also receive a known effective fulgide to control the disease.

To clear the treatment codes, we added a table in the methodology section the revised manuscript as follows:

| Name | Pathogen inoculation | Treatment applied |
| --- | --- | --- |
| Absolute control or Untreated control (AC) | No | No |
| Negative control or inoculated control (NC) | Yes | No |
| Positive control (PC) | Yes | Yes, fungicide as known effective treatment |
| Seed priming (SP) | Yes | Yes, PR5 as seed priming |
| Seedling priming (SeP) | Yes | Yes, PR5 as seedling priming |
| Bacterial culture filtrate (BCF) | Yes | Yes, PR5 culture filtrate as foliar application |
| SP + BCF | Yes | Yes, combined application of PR5 as seed priming and BCF foliar application |
| SeP + BCF | Yes | Yes, combined application of PR5 as seedling priming and BCF foliar application |

The descriptive nomenclature is used throughout the manuscript as per the suggestion.

**Reviewers' comments 7:** The conclusion (Lines 368-377) reads too much like a second abstract, merely rehashing the results. A conclusion should synthesize the study's broader implications and practical applications in sustainable agriculture without repeating detailed data points.
**Response7:** Conclusion is revised according to the suggestion.

**Reviewers' comments 8:** Ensure that the meaning of the error bars is explicitly stated in the general methodology (Section 2.7), not just in the figure captions.

**Response 8:** The meaning of error bars is already mentioned in subsection 2.7 in Methodology in Line 188 as " The results were expressed as the mean ± standard error of mean (n = 3).”

Please consider it.

**9. Reviewers' comments 9:** While generally readable, the manuscript contains minor grammatical inconsistencies and awkward phrasing (e.g., Line 121: "seedlings... are soaked", tense shifts). A final proofread is recommended to ensure consistent past-tense usage for methodologies and results.
**Response 9:** The whole manuscript is rechecked for English language focusing on grammar and typos. Use of past tense was ensured throughout the manuscript. Line 121 is corrected for errors in tense.
