# Supplementary material for: Biocontrol of rice blast by Pseudomonas mosselii PR5 through seed priming and foliar application reduces reliance on chemical pesticides
Source: PLoS One. 2026 Jun 18;21(6):e0351650. doi: 10.1371/journal.pone.0351650 (PMC13278436; doi:10.1371/journal.pone.0351650)
Supplement: Peer review history

# Peer review: Decision Letter 0

**DOI:** 10.1371/journal.pone.0351650.r001

**Authors:** Sofia Isabel Almeida Pereira

16 Apr 2026

PONE-D-25-67322

Biocontrol of rice blast by Pseudomonas mosselii PR5 through seed priming and foliar application reduces reliance on chemical pesticides

PLOS One

Dear Dr. Sultana,

Thank you for submitting your manuscript to PLOS ONE. After careful consideration, we feel that it has merit but does not fully meet PLOS ONE’s publication criteria as it currently stands. Therefore, we invite you to submit a revised version of the manuscript that addresses the points raised during the review process.

Please submit your revised manuscript by May 31 2026 11:59PM. If you will need more time than this to complete your revisions, please reply to this message or contact the journal office at plosone@plos.org. When you're ready to submit your revision, log on to https://www.editorialmanager.com/pone/ and select the 'Submissions Needing Revision' folder to locate your manuscript file.

Please include the following items when submitting your revised manuscript:

- A letter that responds to each point raised by the academic editor and reviewer(s). You should upload this letter as a separate file labeled 'Response to Reviewers'.
- A marked-up copy of your manuscript that highlights changes made to the original version. You should upload this as a separate file labeled 'Revised Manuscript with Track Changes'.
- An unmarked version of your revised paper without tracked changes. You should upload this as a separate file labeled 'Manuscript'.

If you would like to make changes to your financial disclosure, please include your updated statement in your cover letter. Guidelines for resubmitting your figure files are available below the reviewer comments at the end of this letter.

If applicable, we recommend that you deposit your laboratory protocols in protocols.io to enhance the reproducibility of your results. Protocols.io assigns your protocol its own identifier (DOI) so that it can be cited independently in the future. For instructions see: https://journals.plos.org/plosone/s/submission-guidelines#loc-laboratory-protocols. Additionally, PLOS ONE offers an option for publishing peer-reviewed Lab Protocol articles, which describe protocols hosted on protocols.io. Read more information on sharing protocols at https://plos.org/protocols?utm_medium=editorial-email&utm_source=authorletters&utm_campaign=protocols.

As the corresponding author, your ORCID iD is verified in the submission system and will appear in the published article. PLOS supports the use of ORCID, and we encourage all coauthors to register for an ORCID iD and use it as well. Please encourage your coauthors to verify their ORCID iD within the submission system before final acceptance, as unverified ORCID iDs will not appear in the published article. *Only*  the individual author can complete the verification step; PLOS staff *cannot*  verify ORCID iDs on behalf of authors.

We look forward to receiving your revised manuscript.

Kind regards,

Sofia Isabel Almeida Pereira

Academic Editor

PLOS One

Journal Requirements:

When submitting your revision, we need you to address these additional requirements.

1. Please ensure that your manuscript meets PLOS ONE's style requirements, including those for file naming. The PLOS ONE style templates can be found at

https://journals.plos.org/plosone/s/file?id=wjVg/PLOSOne_formatting_sample_main_body.pdf and

https://journals.plos.org/plosone/s/file?id=ba62/PLOSOne_formatting_sample_title_authors_affiliations.pdf

2. Please note that PLOS One has specific guidelines on code sharing for submissions in which author-generated code underpins the findings in the manuscript. In these cases, we expect all author-generated code to be made available without restrictions upon publication of the work. Please review our guidelines at https://journals.plos.org/plosone/s/materials-and-software-sharing#loc-sharing-code and ensure that your code is shared in a way that follows best practice and facilitates reproducibility and reuse.

3. Thank you for stating the following financial disclosure:

"The research was funded by Ministry of Education (MoE), Government of Peoples Republic of Bangladesh (Grant number LS20211677)"

Please state what role the funders took in the study.  If the funders had no role, please state: "The funders had no role in study design, data collection and analysis, decision to publish, or preparation of the manuscript."

If this statement is not correct you must amend it as needed.

Please include this amended Role of Funder statement in your cover letter; we will change the online submission form on your behalf.

4. When completing the data availability statement of the submission form, you indicated that you will make your data available on acceptance. We strongly recommend all authors decide on a data sharing plan before acceptance, as the process can be lengthy and hold up publication timelines. Please note that, though access restrictions are acceptable now, your entire data will need to be made freely accessible if your manuscript is accepted for publication. This policy applies to all data except where public deposition would breach compliance with the protocol approved by your research ethics board. If you are unable to adhere to our open data policy, please kindly revise your statement to explain your reasoning and we will seek the editor's input on an exemption. Please be assured that, once you have provided your new statement, the assessment of your exemption will not hold up the peer review process.

5. If the reviewer comments include a recommendation to cite specific previously published works, please review and evaluate these publications to determine whether they are relevant and should be cited. There is no requirement to cite these works unless the editor has indicated otherwise.

Additional Editor Comments (if provided):

The authors are required to revise the manuscript in accordance with the reviewers’ comments, with particular emphasis on strengthening the methodological aspects.

[Note: HTML markup is below. Please do not edit.]

Reviewers' comments:

Reviewer's Responses to Questions

**Comments to the Author**

1. Is the manuscript technically sound, and do the data support the conclusions?

The manuscript must describe a technically sound piece of scientific research with data that supports the conclusions. Experiments must have been conducted rigorously, with appropriate controls, replication, and sample sizes. The conclusions must be drawn appropriately based on the data presented.

Reviewer #1: Yes

Reviewer #2: Yes

**********

2. Has the statistical analysis been performed appropriately and rigorously?

Reviewer #1: Yes

Reviewer #2: Yes

**********

3. Have the authors made all data underlying the findings in their manuscript fully available?

The PLOS Data policy requires authors to make all data underlying the findings described in their manuscript fully available without restriction, with rare exception (please refer to the Data Availability Statement in the manuscript PDF file). The data should be provided as part of the manuscript or its supporting information, or deposited to a public repository. For example, in addition to summary statistics, the data points behind means, medians and variance measures should be available. If there are restrictions on publicly sharing data—e.g. participant privacy or use of data from a third party—those must be specified.

Reviewer #1: Yes

Reviewer #2: Yes

**********

4. Is the manuscript presented in an intelligible fashion and written in standard English?

PLOS ONE does not copyedit accepted manuscripts, so the language in submitted articles must be clear, correct, and unambiguous. Any typographical or grammatical errors should be corrected at revision, so please note any specific errors here.

Reviewer #1: Yes

Reviewer #2: Yes

**********

5. Review Comments to the Author

Please use the space provided to explain your answers to the questions above. You may also include additional comments for the author, including concerns about dual publication, research ethics, or publication ethics. (Please upload your review as an attachment if it exceeds 20,000 characters)

Reviewer #1: This study addresses a relevant and important topic: the search for sustainable alternatives to chemical fungicides for controlling rice blast, a major threat to global food security. The experimental design is comprehensive, testing multiple application methods of the biocontrol agent Pseudomonas mosselii PR5 across three susceptible rice genotypes. The results demonstrate clear potential for PR5, particularly the combination treatments (SP+BCF and SeP+BCF), in reducing disease severity and promoting plant growth and yield. The manuscript is generally well-structured, but several critical flaws in methodology, data presentation, and interpretation must be addressed before it can be considered for publication.

The phrase "including an absolute control, a pathogen-inoculated control, a fungicide control, and five PR5 application modes" is not clear. It is not clear which control group receives the pathogen and which does not.

The term "seedling priming" (SeP) is introduced but is not a standard term in the literature. "Seed priming" is common

Bacterial culture filtrate (BCF)

Does it refer to a spray of the liquid growth medium containing bacterial metabolites but no live cells? Or is it a suspension of live bacteria?

The statement "SP+BCF achieved the highest yield and outperformed the fungicide in disease suppression" is main conclusion. However, the abstract does not provide the actual disease severity data (e.g., "SP+BCF reduced disease severity by X% compared to the pathogen control, compared to Y% for the fungicide").

For example, "SP+BCF produced the lowest AUPDC in V1 and V3" – how much lower? And crucially, how did it compare to the fungicide in those varieties? Was the difference statistically significant?

The final sentence of the results section ("SP+BCF achieved the highest yield and outperformed the fungicide in disease suppression") largely repeats the point made earlier about SP+BCF producing the lowest AUPDC and achieving the highest yield

The introduction should articulate the specific knowledge gap that this study aims to fill.

The introduction should set up the hypothesis that different application methods (priming vs. foliar) might have different effects, and that combining them could be synergistic. Currently, the introduction presents PR5 as a promising agent but doesn't foreshadow the central question of the paper: What is the best way to use it?

Cite some recent studies in the introduction https://doi.org/10.1016/j.bcab.2020.101729, https://doi.org/10.1002/aoc.5190, https://doi.org/10.1016/j.micpath.2024.107084

The treatment list in lines 106-109 is confusing and contains an error. It states: "T0, absolute control; T1, negative control; T2, positive control (commercial fungicide foliar application); ... T6, seed priming + BCF foliar; and 76, seedling priming + BCF foliar application." Later, in lines 128-132, it mentions a fungicide sprayed in "treatments T1". This is a major inconsistency. Which one is the fungicide treatment? A "negative control" typically receives no treatment and no pathogen, while a "positive control" receives a known effective treatment (like a fungicide) and the pathogen. The authors must clarify the treatment codes and ensure they are used consistently throughout the manuscript, including in all tables and figures.

To properly compare the biocontrol agent to the chemical standard, there must be a treatment that receives the pathogen and is sprayed with the fungicide but does not receive any PR5 treatment. As described, it's impossible to separate the effect of the fungicide from the potential confounding effects of PR5 if the fungicide was applied to the same plants as PR5 treatments.

The method describes the pathogen stock as a "paper disc" (Line 143). This is an unusual and non-standard method for long-term storage of M. oryzae. Typically, cultures are stored on agar slants, in sterile soil, or on filter paper in a dried state. The phrase "presented as a paper disc" is unclear.

Figure 1-3 and figure 9 the figures quality is poor these must be readable.

Reviewer #2: The manuscript investigates the biocontrol efficacy of the endophytic bacterium Pseudomonas mosselii PR5 against rice blast (Magnaporthe oryzae) across three susceptible rice genotypes. The research assesses different application techniques—such as seed priming, seedling priming, and bacterial culture filtrate (BCF) foliar spray—and contrasts their efficacy with that of a commercial fungicide. The findings demonstrate that combined applications, especially SP+BCF and SeP+BCF, significantly mitigate disease severity (PDI and AUDPC) while concurrently promoting plant growth and yield metrics, often surpassing the efficacy of chemical fungicides.

The study addresses an important issue in sustainable agriculture and plant pathology, fitting perfectly within the broad scope of PLOS ONE. The dual-action assessment of PR5 (growth promotion and disease suppression) is commendable. However, I have identified several critical methodological gaps, instances of confusing terminology, and a significant overreach in the Discussion section, in which prior findings are conflated with current data. Before considering the manuscript for publication, the authors should address these issues.

Section 2.2 details the pot preparation but completely omits the statistical design of the experiment. Was this a Completely Randomized Design (CRD) or a Randomized Complete Block Design (RCBD)? Furthermore, the authors note in the Discussion that pots were kept 25 cm apart in natural field conditions and that T0 (absolute control) pots were "physically separated by polyethylene sheets." This crucial methodological information should be included in Section 2.2, rather than being presented as an afterthought in the Discussion to clarify anomalous data. Please detail the exact spatial arrangement, post-inoculation environmental conditions, and randomization protocol.

In the Discussion, the authors state that PR5 produces IAA, solubilizes phosphorus, and produces siderophores and HCN. None of these biochemical assays were conducted or presented in the current manuscript. While these may be established traits of PR5 from the authors' previous works, the current phrasing makes it appear as though these mechanisms were proven in this specific study. The authors must strictly separate their current phenotypic/disease findings from previous in vitro characterizations. Please revise these sections.

In Lines 132-135, the authors mention centrifuging the bacterial solution (OD600 = 1) and mixing the supernatant with CMC for foliar application. Later, in line 225, the solution is referred to as a "bacterial cell-free (BCF)" application. Was this supernatant passed through a microbiological filter (e.g., 0.22 µm) to guarantee it was entirely cell-free? If no filtration was performed, viable cells likely remained in the supernatant, making the term "cell-free" scientifically inaccurate. Please clarify the exact sterilization/filtration protocol. If it was not filtered, the terminology must be corrected throughout the manuscript.

In Lines 124-125, T1 (pathogen only) is labeled the "negative control" and T2 (fungicide) is labeled the "positive control." In plant pathology, a pathogen-only treatment is typically considered a positive control for disease development. To avoid reader confusion, I recommend utilizing descriptive nomenclature throughout the manuscript.

The conclusion (Lines 368-377) reads too much like a second abstract, merely rehashing the results. A conclusion should synthesise the study's broader implications and practical applications in sustainable agriculture without repeating detailed data points.

Ensure that the meaning of the error bars is explicitly stated in the general methodology (Section 2.7), not just in the figure captions.

While generally readable, the manuscript contains minor grammatical inconsistencies and awkward phrasing (e.g., Line 121: "seedlings... are soaked", tense shifts). A final proofread is recommended to ensure consistent past-tense usage for methodologies and results.

**********

6. PLOS authors have the option to publish the peer review history of their article (what does this mean?). If published, this will include your full peer review and any attached files.

If you choose “no”, your identity will remain anonymous but your review may still be made public.

**Do you want your identity to be public for this peer review?**  For information about this choice, including consent withdrawal, please see our Privacy Policy.

Reviewer #1: No

Reviewer #2: **Yes:**  HARUN BEKTAŞ

**********

[NOTE: If reviewer comments were submitted as an attachment file, they will be attached to this email and accessible via the submission site. Please log into your account, locate the manuscript record, and check for the action link "View Attachments". If this link does not appear, there are no attachment files.]

To ensure your figures meet our technical requirements, please review our figure guidelines: https://journals.plos.org/plosone/s/figures

You may also use PLOS’s free figure tool, NAAS, to help you prepare publication quality figures: https://journals.plos.org/plosone/s/figures#loc-tools-for-figure-preparation.

NAAS will assess whether your figures meet our technical requirements by comparing each figure against our figure specifications.

# Author comment: Author response to Decision Letter 1

**DOI:** 10.1371/journal.pone.0351650.r002

13 May 2026

Author Response to Reviewers' comments

Reviewer#1:

Reviewers' comments 1: This study addresses a relevant and important topic: the search for sustainable alternatives to chemical fungicides for controlling rice blast, a major threat to global food security. The experimental design is comprehensive, testing multiple application methods of the biocontrol agent Pseudomonas mosselii PR5 across three susceptible rice genotypes. The results demonstrate clear potential for PR5, particularly the combination treatments (SP+BCF and SeP+BCF), in reducing disease severity and promoting plant growth and yield. The manuscript is generally well-structured, but several critical flaws in methodology, data presentation, and interpretation must be addressed before it can be considered for publication.

Response1: Thank you very much for your valuable comments and important suggestion. We believe your comments and suggestions would significantly improve our manuscript. We have addressed all the comments point-by-point below.

Reviewers' comments 2: The phrase "including an absolute control, a pathogen-inoculated control, a fungicide control, and five PR5 application modes" is not clear. It is not clear which control group receives the pathogen and which does not.

Response 2: The absolute control did not receive the blast pathogen. This treatment also did not receive chemical fungicide or PR5 as a biocontrol agent.

All other treatments received blast pathogen. The negative control received the blast pathogen only. In addition to blast pathogen, positive control received chemical fungicide; and the PR5 treatment group received PR5 trough seed priming (SP), seedling priming (SeP), bacterial culture filtrate (BCF); SP+ BCF and SeP + BCF.

To remove the confusion, we have presented the treatments in a tabular form in the methodology section in the revised manuscript as follows:

Name Pathogen inoculation Treatment applied

Absolute control or Untreated control (AC) No No

Negative control or inoculated control (NC) Yes No

Positive control (PC) Yes Yes, fungicide as known effective treatment

Seed priming (SP) Yes Yes, PR5 as seed priming

Seedling priming (SeP) Yes Yes, PR5 as seedling priming

Bacterial culture filtrate (BCF) Yes Yes, PR5 culture filtrate as foliar application

SP + BCF Yes Yes, combined application of PR5 as seed priming and BCF foliar application

SeP + BCF Yes Yes, combined application of PR5 as seedling priming and BCF foliar application

Reviewers' comments 3: The term "seedling priming" (SeP) is introduced but is not a standard term in the literature. "Seed priming" is common

Response 3: We use the term “seed priming” when we primed the seeds of rice with bacterial solution and designated as “SP” in short form. However, when we primed the seedlings then the method is termed as “seedling priming” and designated as “SeP”. Rice seed is used for direct seeding and seedling is used for transplanted cultivation. Therefore, two distinct priming terms viz. ‘seed priming’ and ‘seedling priming’ were used. Often in scientific paper these two terms used equivalently and term as “seed priming” but we used separate term as these two methods were used as two separate treatments in our experiment.

Reviewers' comments 4: Bacterial culture filtrate (BCF)-Does it refer to a spray of the liquid growth medium containing bacterial metabolites but no live cells? Or is it a suspension of live bacteria?

Response 4: In the current experiment bacterial culture filtrate (BCF) refers the supernatant of bacterial liquid culture. Upon centrifuging the liquid culture of bacteria, the supernatant was used as bacterial culture filtrate. It is not suspension of live bacteria.

Reviewers' comments 5: The statement "SP+BCF achieved the highest yield and outperformed the fungicide in disease suppression" is main conclusion. However, the abstract does not provide the actual disease severity data (e.g., "SP+BCF reduced disease severity by X% compared to the pathogen control, compared to Y% for the fungicide").

For example, "SP+BCF produced the lowest AUPDC in V1 and V3" – how much lower? And crucially, how did it compare to the fungicide in those varieties? Was the difference statistically significant?

Response 5: Few numerical data on disease suppression is included to make the statement clearer in the revised manuscript as follows:

In V1 and V3, SP + BCF even outperformed the chemical fungicide in controlling the disease. For instance, at 28th DAI, SP+ BCF reduced the disease severity by 41.46% and 40.57 % compared to negative control and 5.89% and 14.58 % compared to chemical fungicide treatments in V1 and V3, respectively.

Few other numerical data also added in the result section and the texts are marked.

Reviewers' comments 6: The final sentence of the results section ("SP+BCF achieved the highest yield and outperformed the fungicide in disease suppression") largely repeats the point made earlier about SP+BCF producing the lowest AUPDC and achieving the highest yield

Response 6: The final sentence of the result section actually expressed the superior performance of SP + BCF over chemical fungicide in yield and yield attributes. The earlier sentence in disease matrix part, was highlighted the superior performance of SP + BCF in disease control over all other treatments, especially over chemical fungicide.

However, to avoid the similarity in write up, one line in disease subsection in result is revised as follows:

In V1 and V3, SP + BCF even outperformed the chemical fungicide in controlling the disease. For instance, at 28th DAI, SP+ BCF reduced the disease severity by 41.46% and 40.57 % compared to negative control and 5.89% and 14.58 % compared to chemical fungicide treatments in V1 and V3, respectively.

Reviewers' comments 7: The introduction should articulate the specific knowledge gap that this study aims to fill.

Response 7: Introduction is revised according to the suggestion.

Reviewers' comments 8: The introduction should set up the hypothesis that different application methods (priming vs. foliar) might have different effects, and that combining them could be synergistic. Currently, the introduction presents PR5 as a promising agent but doesn't foreshadow the central question of the paper: What is the best way to use it?

Response 8: The Introduction section is revised and the hypothesis that the bacterial efficacy could be different based on application method.

Reviewers' comments 9: Cite some recent studies in the introduction https://doi.org/10.1016/j.bcab.2020.101729, https://doi.org/10.1002/aoc.5190, https://doi.org/10.1016/j.micpath.2024.107084

Response 9: The mentioned papers are included as citation in Introduction section.

Reviewers' comments 10: The treatment list in lines 106-109 is confusing and contains an error. It states: "T0, absolute control; T1, negative control; T2, positive control (commercial fungicide foliar application); ... T6, seed priming + BCF foliar; and 76, seedling priming + BCF foliar application." Later, in lines 128-132, it mentions a fungicide sprayed in "treatments T1". This is a major inconsistency. Which one is the fungicide treatment? A "negative control" typically receives no treatment and no pathogen, while a "positive control" receives a known effective treatment (like a fungicide) and the pathogen. The authors must clarify the treatment codes and ensure they are used consistently throughout the manuscript, including in all tables and figures.

Response10: Thank you very much for your valuable suggestion.

In our case we referred the treatment that receive no pathogen and no other chemical or biological agent a “absolute control”. The negative control is referred to the treatment that received pathogen but no fungicide or biocontrol agent and the positive control referred to the treatment that received the pathogen and a known fungicide as you mentioned.

There was a Typo in line number 128 (T1 was written instead of T2) that we have corrected in the revised manuscript.

The whole manuscript including all the figures are checked and revised for the consistency of the data code as per the suggestion. The clarification of the treatment code also given with each figure caption.

To clear the treatment codes, we added a table in the methodology section the revised manuscript which is already added in the response 2.

Reviewers' comments 11: To properly compare the biocontrol agent to the chemical standard, there must be a treatment that receives the pathogen and is sprayed with the fungicide but does not receive any PR5 treatment. As described, it's impossible to separate the effect of the fungicide from the potential confounding effects of PR5 if the fungicide was applied to the same plants as PR5 treatments.

Response11: Thank you very much for your valuable suggestion. We do agree with you.

It is noted that we did not use chemical fungicide and PR5 in same treatment. No plants received both chemical fungicide and PR5 in any form of application. The only treatment that received chemical fungicide is the “positive control”. The other plants from T3-T7 received PR5 in different mode of applications. Treatments T0 and T1 did not receive chemical fungicide or PR5. There was a Typo in line number 128 (T1 was written instead of T2) that we have corrected now.

12. Reviewers' comments 12: The method describes the pathogen stock as a "paper disc" (Line 143). This is an unusual and non-standard method for long-term storage of M. oryzae. Typically, cultures are stored on agar slants, in sterile soil, or on filter paper in a dried state. The phrase "presented as a paper disc" is unclear.

Response12: We agree that the phrase “presented as a paper disc” was unclear. The pathogen isolate was not stored as an ordinary paper disc; rather, it was preserved as a dried sterile filter paper disc (round piece of dried sterile filter paper) colonized by Magnaporthe oryzae. This is a recognized method for maintaining M. oryzae isolates for long-term storage.

The sentence is revised to make it clearer in the revised manuscript as follow:

The stock culture of Magnaporthe oryzae was preserved as a dried sterile filter paper disc colonized with the fungus and was revived on potato sucrose agar medium before inoculum preparation.

Reviewers' comments 13: Figure 1-3 and figure 9, the figures quality is poor these must be readable.

Response13: Figures 1–3 and Figure 9 have been reconstructed to improve image quality, and all other figures were carefully checked and revised for consistent figure captions.

Please be noted that, the experimental design involved a complex setup comprising three rice genotypes and eight treatments. Since the data were analyzed using two-way ANOVA, two sets of lettering were required to indicate statistical significance, which made the figures appear crowded and reduced the possible font size. Nevertheless, we adjusted the lettering and formatting to the maximum readable size that could be accommodated within each figure while maintaining clarity and completeness of the statistical information. Orientation of the figures are also changed and rearranged to increase the clarity.

Reviewer #2:

1. Reviewers' comments 1: The manuscript investigates the biocontrol efficacy of the endophytic bacterium Pseudomonas mosselii PR5 against rice blast (Magnaporthe oryzae) across three susceptible rice genotypes. The research assesses different application techniques—such as seed priming, seedling priming, and bacterial culture filtrate (BCF) foliar spray—and contrasts their efficacy with that of a commercial fungicide. The findings demonstrate that combined applications, especially SP+BCF and SeP+BCF, significantly mitigate disease severity (PDI and AUDPC) while concurrently promoting plant growth and yield metrics, often surpassing the efficacy of chemical fungicides.

The study addresses an important issue in sustainable agriculture and plant pathology, fitting perfectly within the broad scope of PLOS ONE. The dual-action assessment of PR5 (growth promotion and disease suppression) is commendable. However, I have identified several critical methodological gaps, instances of confusing terminology, and a significant overreach in the Discussion section, in which prior findings are conflated with current data. Before considering the manuscript for publication, the authors should address these issues.

Response 1: Thank you very much for your constructive comments and suggestions that will be helpful to improve our manuscript. We replied to each comments point-by-point below and corrections has made in the manuscript as and where necessary.

Reviewers' comments 2: Section 2.2 details the pot preparation but completely omits the statistical design of the experiment. Was this a Completely Randomized Design (CRD) or a Randomized Complete Block Design (RCBD)?

Response 2: Thank you for your valuable suggestion.

The experimental design was Completely Randomized Design (CRD). We included the statistical design in the corrected manuscript in Section 2.2.

Reviewers' comments 3: Furthermore, the authors note in the Discussion that pots were kept 25 cm apart in natural field conditions and that T0 (absolute control) pots were "physically separated by polyethylene sheets." This crucial methodological information should be included in Section 2.2, rather than being presented as an afterthought in the Discussion to clarify anomalous data. Please detail the exact spatial arrangement, post-inoculation environmental conditions, and randomization protocol.

Response 3: Actually, the pots that received the blast pathogen was physically separated when blast pathogen was inoculated. As all other treatments except absolute control received blast pathogen, so all the pots except T0 treatment, were taken into a controlled chamber that was separated and isolated. However, after successful disease development the pots were again kept into the same place as they were before.

The spatial arrangement, post-inoculation environmental conditions, and randomization protocol is included in the revised manuscript.

Reviewers' comments 4: In the Discussion, the authors state that PR5 produces IAA, solubilizes phosphorus, and produces siderophores and HCN. None of these biochemical assays were conducted or presented in the current manuscript. While these may be established traits of PR5 from the authors' previous works, the current phrasing makes it appear as though these mechanisms were proven in this specific study. The authors must strictly separate their current phenotypic/disease findings from previous in vitro characterizations. Please revise these sections.

Response 4: The mentioned section (marked as red) in the Discussion is revised to remove any confusion about the finding of current study and the findings of our previous study about the isolate as follows:

The mechanism of growth improvement by PR5 was reported in earlier studies, where this bacterium was found capable of producing significant amounts of indole-3 acetic acid (IAA), fixing atmospheric nitrogen, solubilize insoluble phosphorus, Fe, zinc, and silicon [36-37].

Reviewers' comments 5: In Lines 132-135, the authors mention centrifuging the bacterial solution (OD600 = 1) and mixing the supernatant with CMC for foliar application. Later, in line 225, the solution is referred to as a "bacterial cell-free (BCF)" application. Was this supernatant passed through a microbiological filter (e.g., 0.22 µm) to guarantee it was entirely cell-free? If no filtration was performed, viable cells likely remained in the supernatant, making the term "cell-free" scientifically inaccurate. Please clarify the exact sterilization/filtration protocol. If it was not filtered, the terminology must be corrected throughout the manuscript.

Response 5: We actually used the term bacterial culture filtrate (BCF) in the treatment. The bacterial liquid culture solution was centrifuged and the supernatant was filtered through 0.22 µm syringe filter and used as BCF for foliar application by mixing with CMC.

However, the line 225 (L- 249 in the revised manuscript) is corrected as “bacterial culture filtrate (BCF)” to remove any inconsistency in writing or confusion.

Reviewers' comments 6: In Lines 124-125, T

Attachment Submitted filename: Response to reviewers comments PLOS ONE.docx

# Peer review: Decision Letter 1

**DOI:** 10.1371/journal.pone.0351650.r003

**Authors:** Sofia Isabel Almeida Pereira, Sofia Isabel Almeida Pereira

1 Jun 2026

Biocontrol of rice blast by Pseudomonas mosselii PR5 through seed priming and foliar application reduces reliance on chemical pesticides

PONE-D-25-67322R1

Dear Dr. Sultana,

We’re pleased to inform you that your manuscript has been judged scientifically suitable for publication and will be formally accepted for publication once it meets all outstanding technical requirements.

Within one week, you’ll receive an e-mail detailing the required amendments. When these have been addressed, you’ll receive a formal acceptance letter and your manuscript will be scheduled for publication.

An invoice will be generated when your article is formally accepted. Please note, if your institution has a publishing partnership with PLOS and your article meets the relevant criteria, all or part of your publication costs will be covered. Please make sure your user information is up-to-date by logging into Editorial Manager at Editorial Manager® and clicking the ‘Update My Information' link at the top of the page. For questions related to billing, please contact billing support.

If your institution or institutions have a press office, please notify them about your upcoming paper to help maximize its impact. If they’ll be preparing press materials, please inform our press team as soon as possible -- no later than 48 hours after receiving the formal acceptance. Your manuscript will remain under strict press embargo until 2 pm Eastern Time on the date of publication. For more information, please contact onepress@plos.org.

Kind regards,

Sofia Isabel Almeida Pereira

Academic Editor

PLOS One

Additional Editor Comments (optional):

Reviewers' comments:

Reviewer's Responses to Questions

**Comments to the Author**

1. If the authors have adequately addressed your comments raised in a previous round of review and you feel that this manuscript is now acceptable for publication, you may indicate that here to bypass the “Comments to the Author” section, enter your conflict of interest statement in the “Confidential to Editor” section, and submit your "Accept" recommendation.

Reviewer #1: All comments have been addressed

**********

2. Is the manuscript technically sound, and do the data support the conclusions?

The manuscript must describe a technically sound piece of scientific research with data that supports the conclusions. Experiments must have been conducted rigorously, with appropriate controls, replication, and sample sizes. The conclusions must be drawn appropriately based on the data presented.

Reviewer #1: Yes

**********

3. Has the statistical analysis been performed appropriately and rigorously?

Reviewer #1: Yes

**********

4. Have the authors made all data underlying the findings in their manuscript fully available?

The PLOS Data policy requires authors to make all data underlying the findings described in their manuscript fully available without restriction, with rare exception (please refer to the Data Availability Statement in the manuscript PDF file). The data should be provided as part of the manuscript or its supporting information, or deposited to a public repository. For example, in addition to summary statistics, the data points behind means, medians and variance measures should be available. If there are restrictions on publicly sharing data—e.g. participant privacy or use of data from a third party—those must be specified.

Reviewer #1: Yes

**********

5. Is the manuscript presented in an intelligible fashion and written in standard English?

PLOS ONE does not copyedit accepted manuscripts, so the language in submitted articles must be clear, correct, and unambiguous. Any typographical or grammatical errors should be corrected at revision, so please note any specific errors here.

Reviewer #1: Yes

**********

6. Review Comments to the Author

Please use the space provided to explain your answers to the questions above. You may also include additional comments for the author, including concerns about dual publication, research ethics, or publication ethics. (Please upload your review as an attachment if it exceeds 20,000 characters)

Reviewer #1: (No Response)

**********

7. PLOS authors have the option to publish the peer review history of their article (what does this mean?). If published, this will include your full peer review and any attached files.

If you choose “no”, your identity will remain anonymous but your review may still be made public.

**Do you want your identity to be public for this peer review?**  For information about this choice, including consent withdrawal, please see our Privacy Policy.

Reviewer #1: No

**********

# Editor report: Acceptance letter

**DOI:** 10.1371/journal.pone.0351650.r004

**Authors:** Sofia Isabel Almeida Pereira, Sofia Isabel Almeida Pereira

PONE-D-25-67322R1

PLOS One

Dear Dr. Sultana,

I'm pleased to inform you that your manuscript has been deemed suitable for publication in PLOS One. Congratulations! Your manuscript is now being handed over to our production team.

At this stage, our production department will prepare your paper for publication. This includes ensuring the following:

* All references, tables, and figures are properly cited

* All relevant supporting information is included in the manuscript submission,

* There are no issues that prevent the paper from being properly typeset

You will receive further instructions from the production team, including instructions on how to review your proof when it is ready. Please keep in mind that we are working through a large volume of accepted articles, so please give us a few days to review your paper and let you know the next and final steps.

Lastly, if your institution or institutions have a press office, please let them know about your upcoming paper now to help maximize its impact. If they'll be preparing press materials, please inform our press team within the next 48 hours. Your manuscript will remain under strict press embargo until 2 pm Eastern Time on the date of publication. For more information, please contact onepress@plos.org.

You will receive an invoice from PLOS for your publication fee after your manuscript has reached the completed accept phase. If you receive an email requesting payment before acceptance or for any other service, this may be a phishing scheme. Learn how to identify phishing emails and protect your accounts at https://explore.plos.org/phishing.

If we can help with anything else, please email us at customercare@plos.org.

Thank you for submitting your work to PLOS ONE and supporting open access.

Kind regards,

PLOS ONE Editorial Office Staff

on behalf of

Dr. Sofia Isabel Almeida Pereira

Academic Editor

PLOS One
