# Supplementary material for: Correction: Biocontrol of rice blast by Pseudomonas mosselii PR5 through seed priming and foliar application reduces reliance on chemical pesticides
Source: PLoS One. 2026 Sep 3;21(9):e0357646. doi: 10.1371/journal.pone.0357646 (PMC13541143; doi:10.1371/journal.pone.0357646)
Supplement: Supplementary file 1 [file pone.0357646.s001.pdf]

PLOS One

RESEARCH ARTICLE

Biocontrol of rice blast by *Pseudomonas mosselii* PR5 through seed priming and foliar application reduces reliance on chemical pesticides

Razia Sultana[1]*, Prinon Saha[1], Shah Mohammad Naimul Islam[2], Mohammad Mahbubul Haque[3], Sourav Biswas Shuvo[1], Md. Mustafijur Rahman Khan[1], Seikh Jafor Ahmed[1]

1 Department of Agricultural Chemistry, Bangladesh Agricultural University, Mymensingh, Bangladesh, **2** Institute of Biotechnology and Genetic Engineering (IBGE), Gazipur Agricultural University, Gazipur, Bangladesh, **3** Division of Plant Pathology, Bangladesh Institute of Nuclear Agriculture (BINA), Mymensingh, Bangladesh

* razs@bau.edu.bd

Check for updates

OPEN ACCESS

Citation: Sultana R, Saha P, Islam SMN, Haque MM, Shuvo SB, Khan MMR, et al. (2026) Biocontrol of rice blast by *Pseudomonas mosselii* PR5 through seed priming and foliar application reduces reliance on chemical pesticides. PLoS One 21(6): e0351650. https://doi.org/10.1371/journal.pone.0351650

Editor: Sofia Isabel Almeida Pereira, Portuguese Catholic University: Universidade Catolica Portuguesa, PORTUGAL

Received: December 19, 2025

Accepted: May 31, 2026

Published: June 18, 2026

Peer Review History: PLOS recognizes the benefits of transparency in the peer review process; therefore, we enable the publication of all of the content of peer review and author responses alongside final, published articles. The editorial history of this article is available here: https://doi.org/10.1371/journal.pone.0351650

Copyright: © 2026 Sultana et al. This is an open access article distributed under the terms of the Creative Commons Attribution License,

Abstract

Rice blast caused by *Magnaporthe oryzae* is a destructive disease that can infect rice at any developmental stage. This study investigated the biocontrol potential of the endophytic bacterium *Pseudomonas mosselii* PR5 against rice blast in comparison with a chemical fungicide. Three blast-susceptible rice genotypes were evaluated under eight treatment combinations, including an absolute control, a pathogen-inoculated control, a fungicide control, and five PR5 application modes: seed priming (SP), seedling priming (SeP), bacterial culture filtrate (BCF) foliar spray, and the combinations SP + BCF and SeP + BCF. All treatments except the absolute control received pathogen inoculation. Both PR5 and the fungicide significantly reduced disease severity across the three genotypes. The pathogen-only treatment consistently recorded the highest percent disease index (PDI) and area under the disease progress curve (AUPDC). Among the bacterial treatments, SP + BCF produced the lowest AUPDC in V1 and V3, while the fungicide performed best in V2. PR5 inoculation also enhanced plant growth and yield. Shoot dry weight increased by 3.29–47.36% compared with the absolute control and by 10.10–107.43% compared with the pathogen-only treatment. Pathogen stress severely reduced root growth, whereas PR5, particularly in the SeP + BCF treatment, increased root biomass by 24.58–69.22%. Significant improvements in yield traits like grains per panicle, effective tillers, and reduced chaffy grains were observed, especially when priming was combined with BCF foliar application. SP + BCF achieved the highest yield and outperformed the fungicide in disease suppression. These results suggest that PR5-based seed or seedling priming combined with BCF foliar application is a promising strategy for sustainable rice blast management.

which permits unrestricted use, distribution, and reproduction in any medium, provided the original author and source are credited.

Data availability statement: All relevant data are contained within the manuscript.

Funding: The research was funded by Ministry of Education (MoE), Government of Peoples Republic of Bangladesh (Grant number LS20211677).

Competing interests: The authors have declared that no competing interests exist.

1 Introduction

Rice is a staple food and is vital for the food security of approximately half of the population globally [1,2]. The production of rice is often hampered by different abiotic stressors such as salinity, drought, heat, cold, etc., and biotic stress imposed by different insects and phytopathogens [3]. Among the biotic stresses, rice blast, caused by *Magnaporthe oryzae* (syn. *Pyricularia oryzae*), is one of the most destructive fungal diseases affecting rice crops and causing 10–30% yield loss each year worldwide [4,5]. The pathogen invades the plant through the leaf surface, damaged rice leaves and panicles, indicating that it harms both vegetative and reproductive stages [6,7]. As the global demand for rice increases, finding sustainable and effective methods to combat rice blast has become critical.

Traditional methods of controlling rice blast often involve the use of chemical fungicides, which, while effective in the short term, come with several challenges [8,9]. The overuse of these chemicals can lead to the development of resistant pathogen strains [10]. Besides, chemical fungicides can harm non-target organisms and also cause severe environmental degradation and leave harmful effects on human health [11]. As a result, reliance on chemical pesticides is increasingly being scrutinized, and there is a growing need for more sustainable and environmentally friendly alternatives [12,13]. Use of a bioagent can be useful in this aspect to reduce the adverse effect of chemical fungicide. Biological control through the use of beneficial microorganisms is gaining attention as part of integrated pest management (IPM) strategies, aiming to reduce chemical pesticide dependence while ensuring effective disease control. These biocontrol agents are generally safer for humans, animals, and the environment [14–16], and they work through natural mechanisms, such as the induction of plant immunity, competition for nutrients, or direct antagonism against pathogens [17–20].

Several microbes have been reported for their plant growth-promoting traits, as well as disease control efficacy [21]. Species of *Bacillus*, *Trichoderma*, *Stenotrophomonas*, *Metarhizium*, and *Beauveria* have shown great efficacy against several biotic stressors, including rice blast [22–26]. In this context, *Pseudomonas* has shown potential as a biocontrol agent against several phytopathogens in different crops [27–29]. *Pseudomonas aeruginosa* FG106 has shown potentiality against potato, tomato and taro pathogens [30]. Phenazine-producing *Pseudomonas* spp. have been reported to work as biocontrol agents against several plant pathogens [31]. *Pseudomonas putida* has been reported as a potential biocontrol agent against rice blast and common bean rust [32,33]. Endophytic *Pseudomonas putida* BP25 produced pyrazines, which were found to be effective against rice blast disease [34].

Pseudomonas mosselii is a ubiquitous and metabolically versatile bacterium which belongs to the *Pseudomonas putida* group. Recent studies indicate that this bacterial endophyte can suppress the growth and spread of *M. oryzae*, thereby mitigating the damage caused by rice blast [35]. Different biochemicals and defence-related gene clusters have been reported to be responsible for the direct antagonism [36,37]. Additionally, the strain appears to induce systemic resistance in plants through several mechanisms [38]. These make *P. mosselii* a promising candidate for integrated pest management strategies aimed at protecting rice crops from blast disease.

Despite the potential of PGPR in plant growth promotion and stress mitigation, its efficacy largely depends on the application methods. Seed priming with PGPRs increased seed germination, improved seedling vigour, and enhanced early stress tolerance by allowing bacteria to colonize seeds before emergence [39,40]. In rice, both seed and seedling priming with endophytic bacteria showed improved root development and uptake of key nutrients such as N, P, K, Fe, and Zn [41]. Foliar application of bioinoculants improved photosynthetic parameters, nutrient accumulation efficiency, root growth, and reduce stress in rice, tomato, maize, grasses, and spinach [42–46]. Thus, combining different PGPR delivery methods in plant systems shows a potential strategy for additive or synergistic gains in yield, nutrient profile, and stress protection.

Pseudomonas mosselii strain PR5 isolated from rice rhizosphere has shown potential as a plant growth promoter with several PGP traits and thereby increased germination, rice growth, yield and nutrient contents, improved post-harvest soil nutrients and protected the rice plants from naturally occurring blast disease [47,48]. However, the potentiality of this native isolate remains unexplored under *M. oryzae* challenged conditions. In the current experiment this native isolate was used in different modes of application to control rice blast in comparison to a chemical fungicide when inoculated by *M. oryzae* in three rice genotypes. The aim of this research is to determine the potentiality of PR5 to suppress rice blast disease in comparison to a chemical fungicide and to find out the best application strategy of this biocontrol agent in rice for better yield.

2 Materials and methods

2.1 Microbial strains and culture conditions

Native isolate *P. mosselii* strain PR5, isolated from rice rhizosphere, was obtained from our laboratory culture collections. The 16S rRNA nucleotide sequencing was deposited to the NCBI GenBank with the accession number MZ540030. The isolate was grown in Nutrient Broth (NB) media supplemented with agar. A loop of bacterial culture was inoculated in liquid NB media and grown for 24–48 hours for liquid culture preparation. The culture was then preserved for further use in a refrigerator at 4°C.

The pathogenic strain of *M. oryzae* BNA02 (MO) was collected from the culture collection of our laboratory, which was previously isolated from a diseased rice plant. The isolate was molecularly characterized and 16S rRNA nucleotide sequence was deposited to the NCBI database with the GenBank accession number PQ278691.

2.2 Pot experiment procedure

Three rice genotypes were used in this study. These are BRRI dhan28, which is a popular rice variety but susceptible to blast, designated as V1; BINA dhan18, which is also susceptible to blast, designated as V2; and a susceptible rice line, US2 (Universal Susceptible line 2), designated as V3. The first two varieties are the released varieties and are available throughout the country for farmers' use, and, therefore, no permission was necessary to use those. The susceptible line US2 was collected from the BINA germplasm collections with the authorized permission for restricted use for research purposes only.

The seeds of the three rice varieties are soaked in 0.5% sodium hypochlorite for 5 minutes and then washed several times with sterile water. Subsequently, the seeds were soaked overnight in PR5 bacterial solution or in water. Then the seeds were placed in wet paper in petridishes for germination. The sprouted seeds were placed in separate plastic pots to raise the seedlings. The seedlings were uprooted from the seedbed at one month of age and were soaked in PR5 solution or distilled water for 12 hours before being transplanted into the pots.

Pots were prepared with 12 kg of field soil in each pot. The soil was mixed with recommended doses of fertilizers for *boro* rice in FRG, 2018 [49]. The PR5 inoculated or uninoculated seedlings were transplanted into the pots, having one seedling per pot. The pots were kept 25 cm apart from each other. The experiment was carried out in Completely Randomized Design (CRD) with three replications. There were 8 treatments, namely T0, absolute control (uninoculated

control); T1, negative control; T2, positive control (commercial fungicide foliar application); T3, seed priming; T4, seedling priming; T5, bacterial culture filtrate (BCF) foliar application; T6, seed priming + BCF foliar; and T7, seedling priming + BCF foliar application. All the treatments except absolute control received blast pathogen. Table 1 shows the detail of the treatments used in this study. The pots were rearranged once in a week to ensure complete randomization. Watering and weeding were done when necessary. The split application of urea was done for the 2nd and 3rd doses at the tillering stage and at the initiation of flowering.

2.3 Chemical fungicide and bacterial foliar applications

P. mosselii PR5 was freshly cultured from the stock and used to prepare liquid culture with an optical density of 1 at 600 nm. The bacterial solution was centrifuged, and the supernatant was mixed with 0.5% carboxymethyl cellulose (CMC) before foliar application as described by Sultana et al. (2024a) [41]. The preventive foliar application of PR5 was done in treatments T5, T6 and T7, whereas commercial fungicide was sprayed in treatment T2 at 35 days after transplanting (DAT) through separate hand sprayers over the rice plants at the rate of 50 mL/pot. Commercial fungicide Amistar Top (Azoxystrobin + Difenoconazole) collected from Syngenta (Bangladesh) Ltd. The solution was prepared by mixing 2 mL fungicide into 1 L distilled water and sprayed at 50 mL/plant at 35 DAT. Another curative spray of *P. mosselii* and commercial fungicide was done at 50 DAT after application of the blast pathogen.

2.4 Inoculum preparation and spraying of blast pathogen

The stock culture of *M. oryzae* (MO) was preserved as a dried, sterile filter paper disc colonized with the fungus and was revived on potato sucrose agar medium before inoculum preparation. The plates were incubated at 25 °C for 12–14 days to promote fungal growth. After the incubation period, the cultivated culture was gently scraped off using a sterilized toothbrush. To induce robust sporulation, the plates were exposed to continuous light for 4–5 days. The conidia, which are the spore structures of the fungus, were then loosened by delicately rubbing the incubated plates with a paintbrush into sterilized distilled water containing 0.01% Tween 20. The resulting spore suspensions were filtered through four layers of gauze mesh to remove any large debris. The concentration of the spore suspension was adjusted to 10^5 conidia/mL using a haemocytometer. The suspension was then sprayed over the rice plants in all the treatments except absolute control (T0) at 42 DAT using a hand sprayer. Before spraying, the plants that were subjected to get the pathogen placed in a closed chamber at approximately 28°C to facilitate infection. The relative humidity was kept between 80% and 90% for successful disease development. This controlled environment was maintained for 7 days, and then the plants were opened in a normal field environment. The pots were returned to the experimental site and arranged in the same set up used before inoculation. During the disease inoculation period, the absolute control pots were kept outside the closed chamber at the experimental site in their original positions.

Table 1. Details of the experimental treatments.

Name	Pathogen inoculation	Treatment applied
Absolute control or Untreated control (AC)	No	No
Negative control or inoculated control (NC)	Yes	No
Positive control (PC)	Yes	Yes, chemical fungicide as known effective treatment.
Seed priming (SP)	Yes	Yes, PR5 as seed priming.
Seedling priming (SeP)	Yes	Yes, PR5 as seedling priming.
Bacterial culture filtrate (BCF) foliar application	Yes	Yes, PR5 culture filtrate as foliar application.
SP + BCF	Yes	Yes, combined application of PR5 as seed priming and BCF foliar application.
SeP + BCF	Yes	Yes, combined application of PR5 as seedling priming and BCF foliar application.

https://doi.org/10.1371/journal.pone.0351650.t001

2.5 Measurement of disease incidence and severity

The disease incidence and severity data were recorded on the 7th, 14th and 21st and 28th days after *M. oryzae* application. The disease incidence was determined by the number of infected tillers among the number of total tillers in each treatment as described by Rafi et al. [50]. From each pot, 10 leaves were randomly chosen, and the disease severity was recorded. A 0–9 scoring was done for the disease spots, followed by the Standard Evaluation System (SES) for rice as developed by the International Rice Research Institute (IRRI) [51]. The PDI was calculated using the formula as described by Baker, 1970 [52], as given below:

$$PDI = \frac{\text{sum of the individual disease rating}}{\text{Total number of leaves examined} \times \text{ Highest rating}} \times 1$$

From the disease severity (%) or PDI values, the area under disease progress curve (AUPDC) at each observation was calculated using the formula [53]:

$$AUPDC = \sum (y_i + y_{i+1})/2 \times (t_{i+1} - t_i)$$

Where:

- y_i = disease severity (%) at i-th observation time.
- y_{i+1} = disease severity (%) at next observation time (i+1).
- t_i = time (days after inoculation or observation date) at i-th observation.
- t_{i+1} = time at next observation (i+1).

2.6 Harvesting and data collection

The rice plants were harvested at the ripening stage when the seeds were golden in color. Data were collected for the plant height, number of tillers, and number of effective or panicle-bearing tillers one day prior to harvesting. The root length, number of grains/panicle, number of chaffy grains/panicles, 1000-grain weight, grain moisture, and fresh and dry weight of roots and shoots were collected just after harvesting was done. To count the number of total grains, filled grains and chaffy grains per panicle, 6 panicles were randomly chosen from each pot, and the number of grains was counted from each panicle separately. Therefore, in total, 18 panicles were considered from each treatment, and the final calculation was done by averaging the data. The diseased leaves with distinct blast spots were also collected, and images were taken.

2.7 Statistical analysis

Data were organized and visualized by the computer package program Microsoft Excel. The statistical software 'R version 3.4.2 was employed for the comprehensive analysis of the data. The results were expressed as the mean ± standard error of the mean (n = 3). To ascertain intra-varietal and inter-varietal differences among the treatments being studied, a two-way analysis of variance (ANOVA) was used. The 'agricolae' package in R version 3.4.2 was then used to do post-hoc analyses using Tukey's test at $P < 0.05$.

2.8 Ethics approval

The experiment did not use any human or animal participants. Microbial work and pathogen inculcation were carried out following approved biosafety protocols.

3 Results

3.1 Growth response of rice as influenced by PR5 application under blast stress

Application of *P. mosselii* PR5 markedly enhanced rice growth compared to the uninoculated control, even under *M. oryzae* (MO)-induced blast stress. Fig 1 shows the visual observation of shoot growth of three varieties of rice at harvest. The pathogen imposed considerable biotic stress, resulting in growth suppression, which was most evident in variety V3 (US2). Nevertheless, both PR5 and the chemical fungicide effectively mitigated the adverse effects of the pathogen and promoted overall plant growth.

MO infection reduced the number of tillers by 18–29% across all varieties relative to the absolute control (Fig 2B). Conversely, chemical fungicide treatment (positive control) compensated for this reduction, increasing tiller numbers by 32% in V1 and 5% in V2. PR5-inoculated treatments also counteracted the pathogen's impact, leading to a 2.35–41.44% increase in tiller number compared to the absolute control and 2.89–82.56% over the negative control across the varieties.

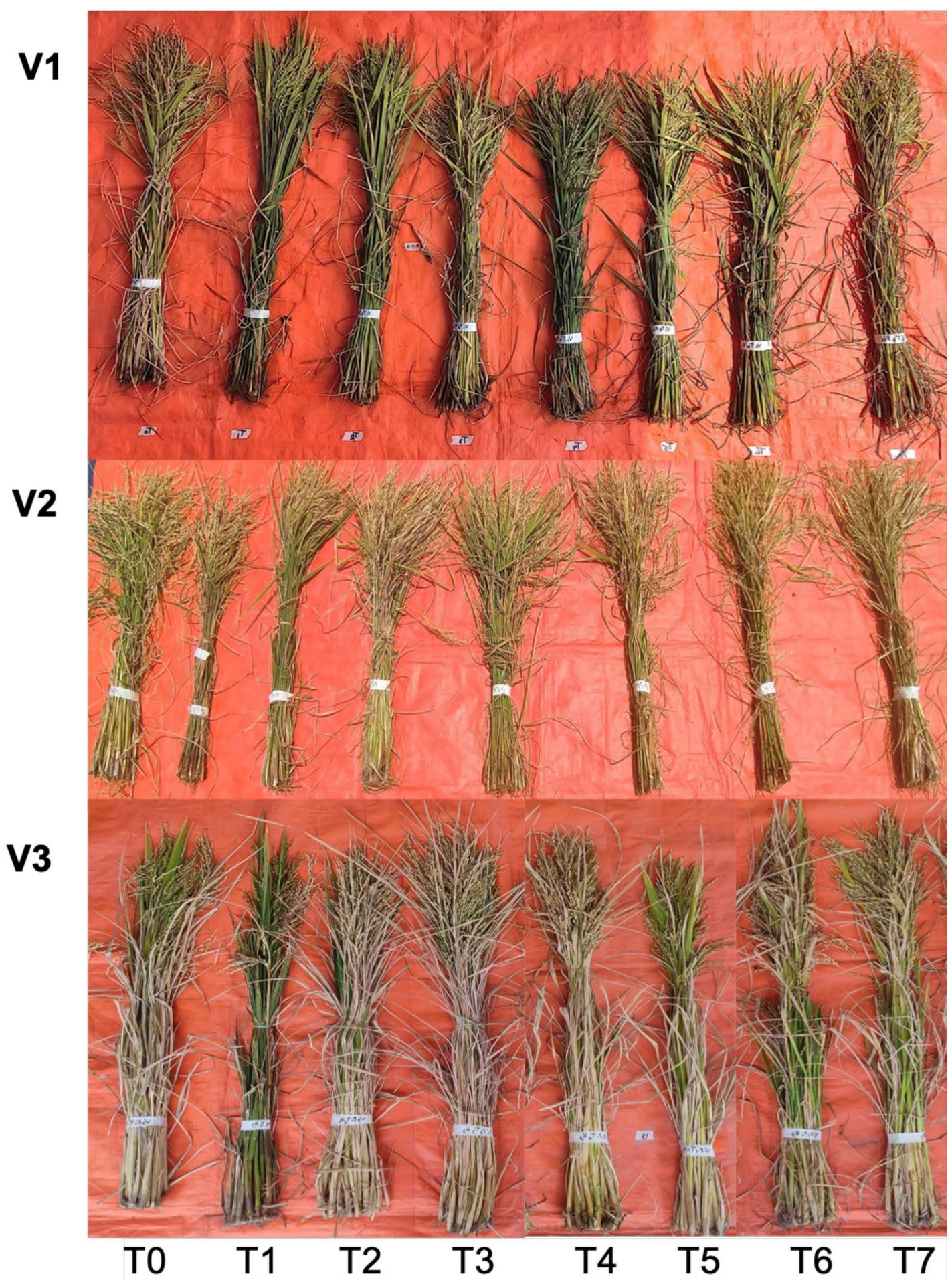

Fig 1. Photographs showing the above ground growth of rice plants of variety 1 (V1), variety 2 (V2) and verity 3 (V3) at harvest as exposed to rice blast pathogen and inoculated by PR5 in different treatments. T0 = absolute control, T1 = negative control, T2 = Positive control, T3 = Seed priming, T4 = Seedling priming, T5 = bacterial culture filtrate (BCF) foliar application, T6 = Seed priming + BCF foliar application and T7 = Seedling priming + BCF foliar application.

https://doi.org/10.1371/journal.pone.0351650.g001

Similarly, effective tillers were reduced by 18–34% compared to the absolute control due to MO infection in negative control treatment. In comparison, the fungicide treatment improved effective tiller numbers by 2.98%, 27%, and 2.59% over the absolute control whereas, it increased the effective tillers by 59.49%; 56.81% and 25.39% over the negative control, in V1, V2, and V3, respectively. PR5 – inoculated treatments also increased the effective tillers by 41.7–60.75%, 17–65.9% and 3.17–41.26% over the negative control in V1, V2, and V3, respectively.

Shoot fresh and dry biomass were also significantly reduced under MO stress (Figs 2C, 2D). However, PR5 treatments effectively compensated for these losses. Notably, seed priming (SP) or seedling priming (SeP) combined with bacterial culture filtrate (BCF) foliar application substantially increased biomass across all varieties, with the effect being most pronounced in V1 and V3. Overall, PR5 application through seed or seedling priming coupled with BCF foliar spraying outperformed the chemical fungicide in all shoot growth parameters. The plant height was not significantly affected due to bacterial application or blast pathogen treatment in V1 and V3. However, a significant reduction in plant height was observed in V2 under MO treatment (Fig 2A).

3.2 Root growth of rice under blast challenged condition

The impact of the rice blast pathogen was more pronounced on rice root growth than on shoot growth. Both fresh and dry root weights were significantly reduced under pathogen infection, with the lowest values observed in the MO-treated plants. Fig 3 revealed the visual observation of root growth. Root length also declined under MO stress in all varieties except V3 (Fig 4A). Compared to the control, fresh root biomass was decreased by 28.14%, 55.12%, and 5.91%, whereas dry root biomass decreased by 33.29%, 60.5%, and 11% in V1, V2, and V3, respectively, under MO treatment (Figs 4B, 4C).

Bacterial inoculation not only mitigated the loss but also enhanced the root biomass. Among the treatments, seedling priming combined with bacterial culture filtrate (BCF) foliar application (SeP + BCF) produced the highest root biomass in all three varieties, next to which was seed priming (SP) + BCF. Compared to the control, seed priming (SP) and seedling priming (SeP) combined with BCF foliar application increased root dry weight by 47.46% and 68.88% in V1, 47.68% and 69.22% in V2, and 25.42% and 24.58% in V3, respectively.

3.3 Leaf blast disease suppression by PR5 under *M. oryzae*-challenged condition

Upon inoculation with the blast pathogen, characteristic leaf blast symptoms were observed in all rice plants. However, disease severity was significantly lower in *P. mosselii* (PR5)-inoculated treatments compared with plants treated only with *M. oryzae* (MO), which exhibited the highest disease severity across all varieties. Fig 5 shows the pictures of representative leaves which show typical symptoms of leaf blast at 7 days after MO inoculation, where it is clear that treatment T1, which received MO only, had the highest disease symptoms in the leaves.

Fig 6 shows that PDI was consistently highest in treatment T1 (MO-inoculated plants) throughout the scoring periods across varieties. In all varieties, disease severity increased sharply from the day of inoculation to the first scoring at 7 days after inoculation (DAI) and then gradually progressed through the following two weeks. In contrast, the absolute control exhibited the lowest disease severity at 7th, 14th, and 21st DAI, but a noticeable rise was recorded at 28th DAI. At this stage, PDI in the absolute control exceeded that of several treatments: higher than CF, SP, SP + BCF, and SeP + BCF in V1 and higher than CF, BCF, SP + BCF, and SeP + BCF in V2 and V3 (Figs 6B, 6C). However, the PDI of absolute control was consistently lower than the negative control at all the data collection points. Among the bacterial treatments, SP + BCF consistently resulted in the lowest disease severity across all three varieties, followed by SeP + BCF, BCF, and SP. In V1 and V3, SP + BCF even outperformed the chemical fungicide in controlling the disease. For instance, at 28th DAI, SP + BCF reduced the disease severity by 41.46% and 40.57% compared to negative control and 5.89% and 14.58% compared to chemical fungicide treatments in V1 and V3, respectively.

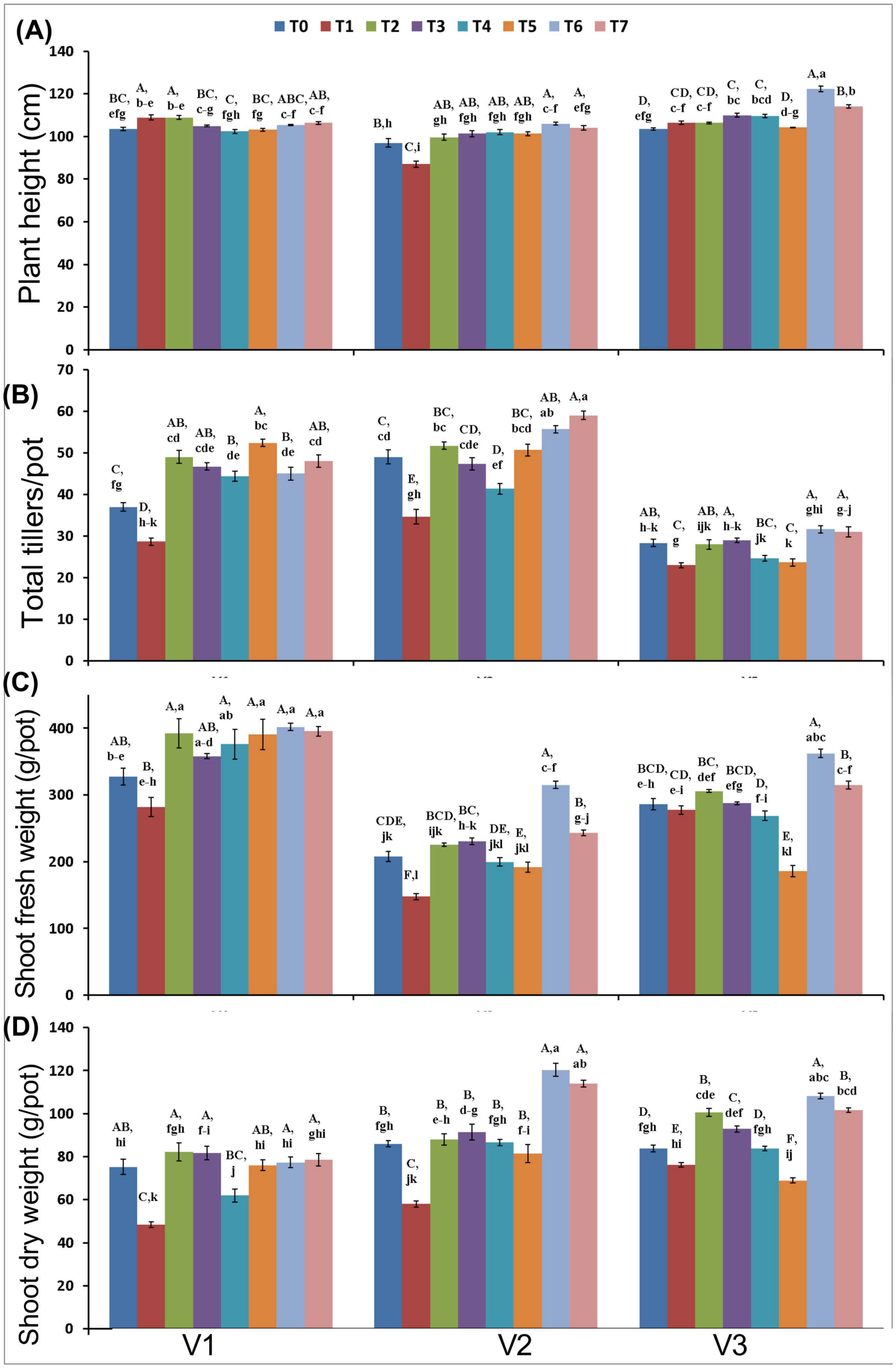

Fig 2. Effect of application of PR5 on rice growth as challenged by *M. oryzae*. (A) Plant height, **(B)** Total tillers, **(C)** Shoot fresh weight, **(D)** Shoot dry weight. Capital letters represent the significant variations among the treatments within the variety and small letters represent the variation among the treatments under three varieties (Tukey's test, $P < 0.05$). Bar represents the mean ± standard error (n = 3). T0 = Absolute control, T1 = Negative control,

T2 = Positive control, T3 = Seed priming, T4 = Seedling priming, T5 = Bacterial culture filtrate (BCF) foliar application, T6 = Seed priming + BCF foliar application and T7 = Seedling priming + BCF foliar application.

https://doi.org/10.1371/journal.pone.0351650.g002

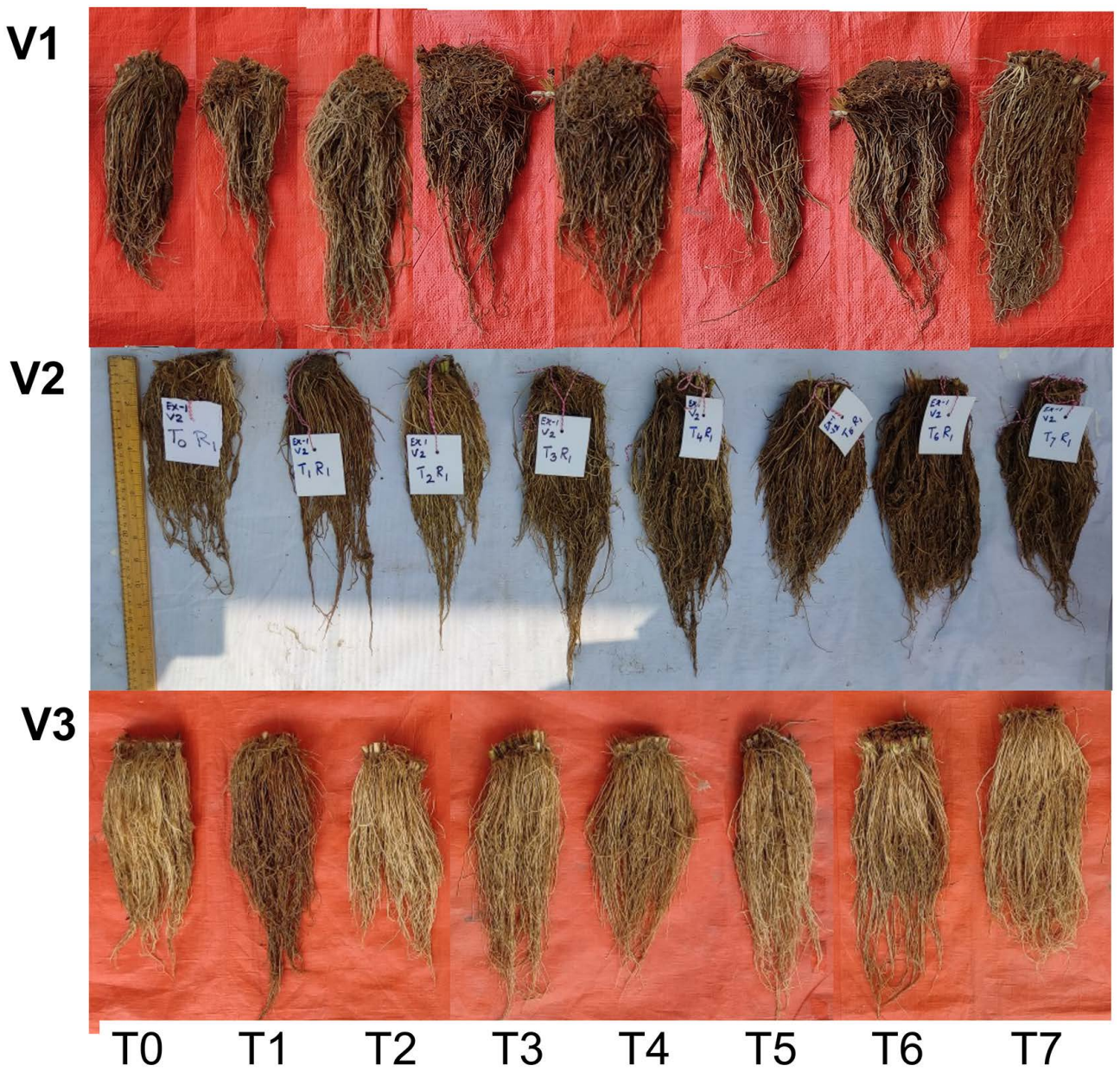

Fig 3. Visual observation of root growth of rice plants of variety I (V1), variety 2 (V2) and verity 3 (V3) at harvest as exposed to rice blast pathogen and inoculated by PR5 in different treatments. T0 = Absolute control, T1 = Negative control, T2 = Positive control, T3 = Seed priming, T4 = Seedling priming, T5 = Bacterial culture filtrate (BCF) foliar application, T6 = Seed priming + BCF foliar application and T7 = Seedling priming + BCF foliar application.

https://doi.org/10.1371/journal.pone.0351650.g003

Similarly, MO-treated plants showed the highest area under the disease progress curve (AUDPC) values in all three varieties (Fig 7). In V1 and V3, AUDPC curves were sharp at 7 DAI and continued to increase steadily until 28 DAI, while in V2, AUDPC rose sharply after 21 DAI. Both bacterial and fungicide treatments effectively reduced AUDPC values, with SP + BCF showing the most consistent suppression across varieties. The chemical fungicide performed comparably to bacterial treatments in V1 and V3, whereas in V2, it achieved slightly greater AUDPC reduction. The lowest AUDPC values were observed in the absolute control at 7, 14, and 21 DAI, with a moderate increase at 28 DAI similar to bacterial treatments. In V3, the AUDPC curve of the absolute control overlapped with those of the bacterial and fungicide treatments, with SP + BCF maintaining the lowest overall disease progression.

3.4 Effect of PR5 on yield attributes and yield of rice under *M. oryzae* applied condition

In all three varieties, application of PR5 increased rice yield compared with the control, even under MO infection. MO treatment markedly impaired the reproductive growth of rice, leading to substantial reductions in yield components. The number of effective tillers (panicles), grains per panicle, total grains per pot, and an increase in chaffy grains per panicle in MO treatment collectively contributed to yield reduction compared with both the absolute control and other treatments across all three rice genotypes (Fig 8A-8D).

Despite pathogen infection, PR5 application enhanced yield traits, particularly the number of effective tillers, which increased by 14.14–27.27% in V1 compared with the control (Fig 8A). In V2 and V3, only SP + BCF and SeP + BCF

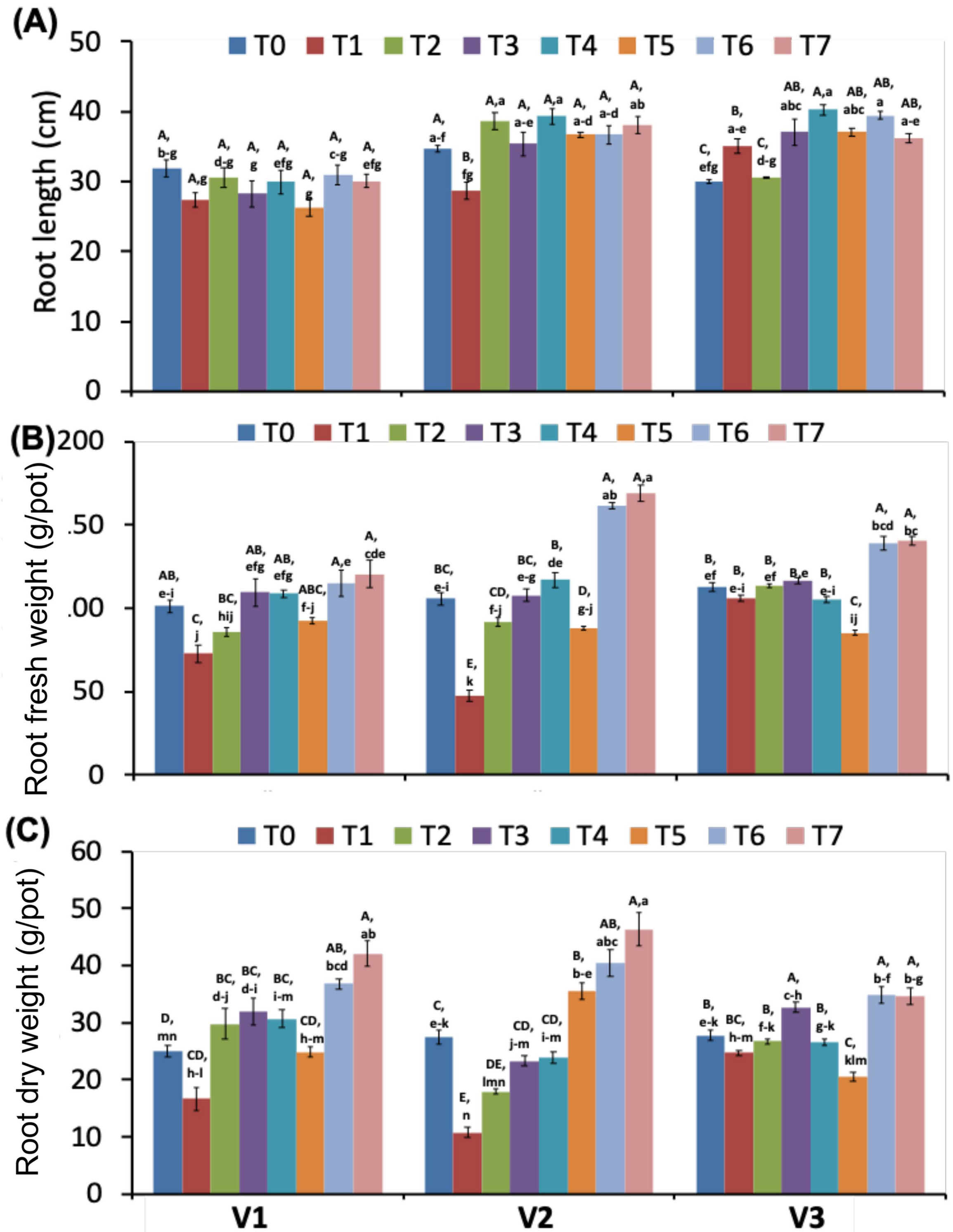

Fig 4. Effect of application of PR5 on root growth of rice in variety I (V1), variety 2 (V2) and verity 3 (V3) as exposed to *M. oryzae*. (A) Root length, **b)** Root fresh weight **(C)** Root dry weight. Capital letters represent the significant variations among the treatments within the variety and small letters represent the variation among the treatments under three varieties (Tukey's test, $P < 0.05$). Bar represents the mean with standard error (n = 3).

T0 = Absolute control, T1 = Negative control, T2 = Positive control, T3 = Seed priming, T4 = Seedling priming, T5 = Bacterial culture filtrate (BCF) foliar application, T6 = Seed priming + BCF foliar application and T7 = Seedling priming + BCF foliar application.

https://doi.org/10.1371/journal.pone.0351650.g004

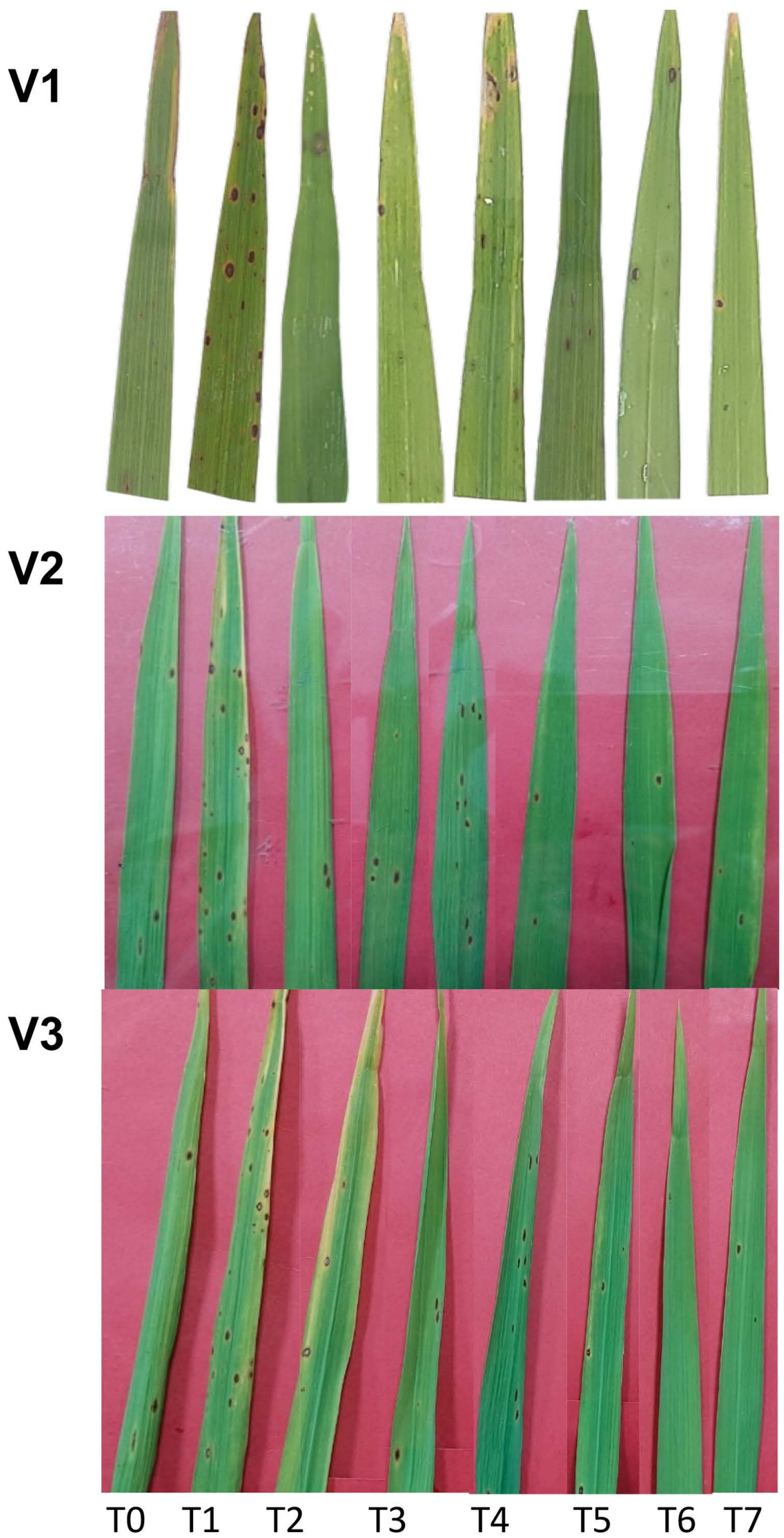

Fig 5. Visual observation of diseased rice leaves in variety I (V1), variety 2 (V2) and variety 3 (V3) at 7th DAI as challenged by *M. oryzae*. T0 = Absolute control, T1 = Negative control, T2 = Positive control, T3 = Seed priming, T4 = Seedling priming, T5 = Bacterial culture filtrate (BCF) foliar application, T6 = Seed priming + BCF foliar application and T7 = Seedling priming + BCF foliar application.

https://doi.org/10.1371/journal.pone.0351650.g005

treatments significantly increased the number of effective tillers, while other bacterial application methods had limited effects. The number of filled grains per panicle increased significantly in V1 and V3 due to PR5 application (Fig 8B). Notably, PR5 inoculation in all treatments reduced the number of chaffy grains compared with both the absolute and

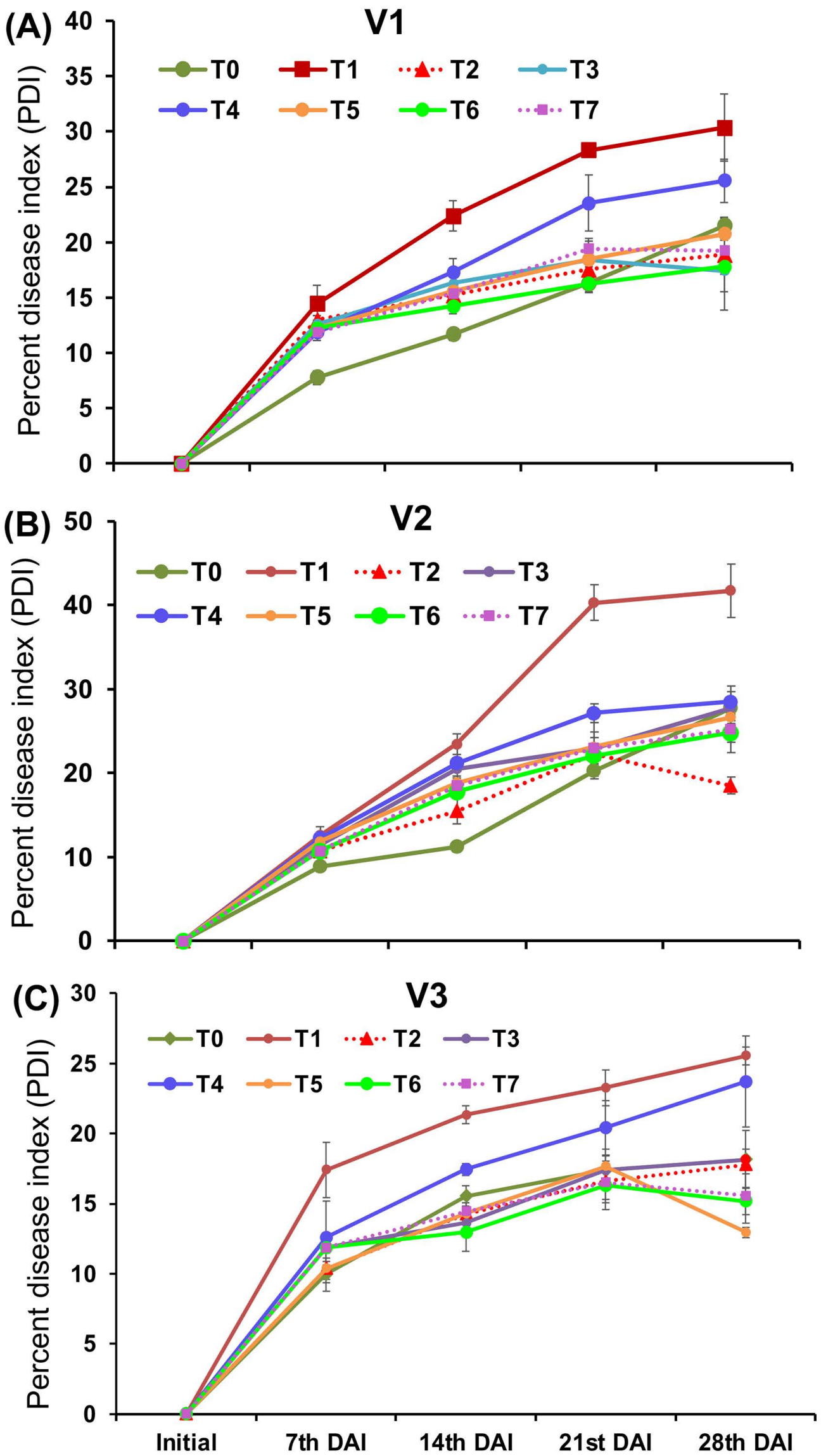

Fig 6. Disease severity expressed by percent disease index (PDI) in rice leaves at 7th, 14th, 21st and 28th DAI as challenged by *M. oryzae* in three varieties. Bar represents the mean ± standard error (n = 3). T0 = Absolute control, T1 = Negative control, T2 = Positive control, T3 = Seed priming, T4 = Seedling priming, T5 = Bacterial culture filtrate (BCF) foliar application, T6 = Seed priming + BCF foliar application and T7 = Seedling priming + BCF foliar application.

https://doi.org/10.1371/journal.pone.0351650.g006

pathogen-inoculated controls, as well as the fungicide-treated plants in V2 and V3 (Fig 8C). The 100-grain weight was increased by the application of PR5 compared to both control and MO-treated plants in V1 and V2 but not in V3 (Fig 8D).

Fig 9A shows the photographic view of the panicles after harvest. The panicle fresh weight was significantly reduced by MO application, and both chemical fungicide and PR5 compensated for the loss. The maximum panicle weight was recorded in the SP + BCF treatment in V2 and V3, while in V1 it was found in the SP + BCF treatment (Fig 9B).

Overall, PR5 inoculation improved grain yield across all rice varieties, with the most pronounced response observed in V2, followed by V1 and V3. In V1 and V3, the highest grain yield was obtained with the SP + BCF treatment, while in V2,

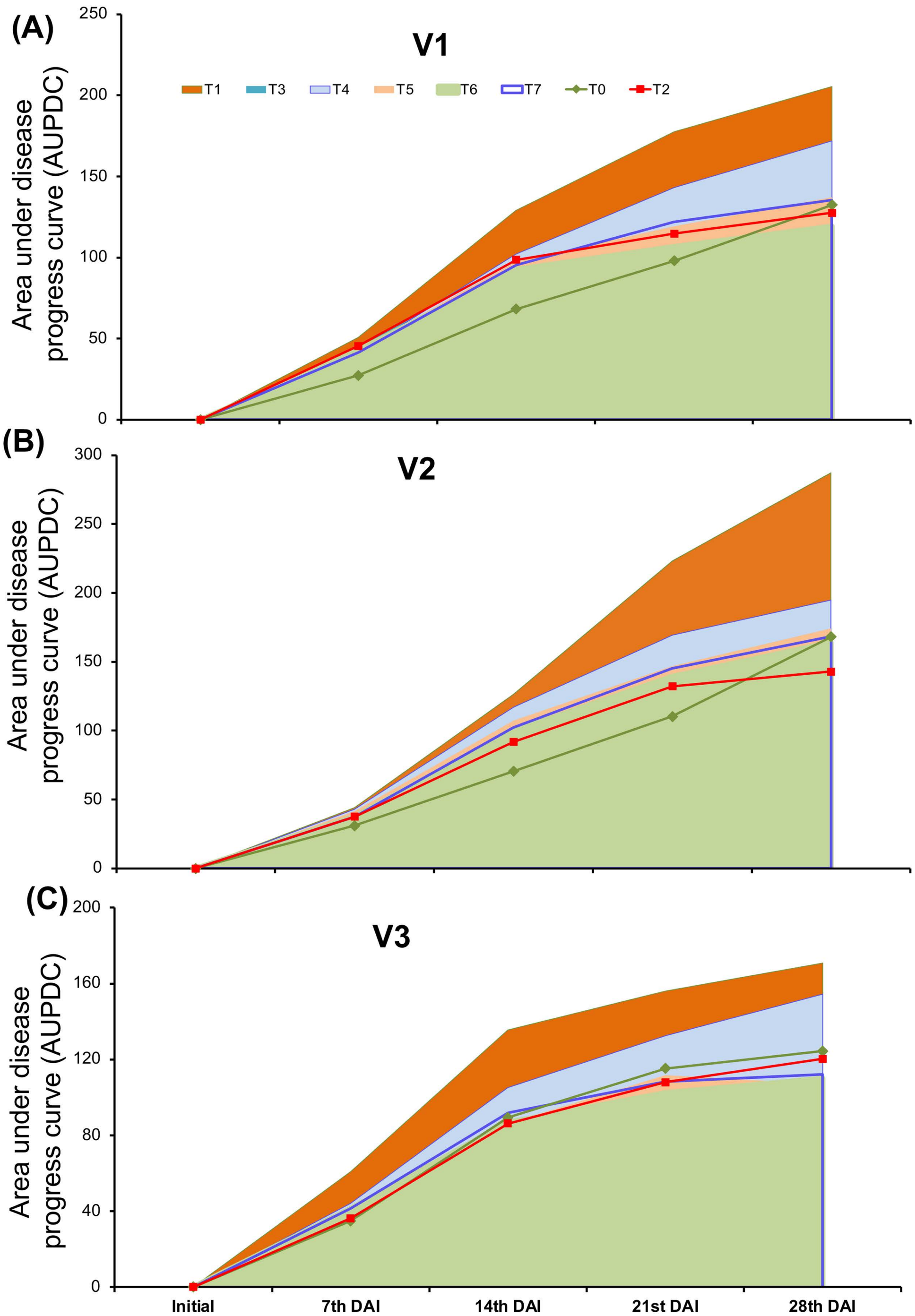

Fig 7. Area under disease progress curve (AUPDC) in rice variety I (V1), variety 2 (V2) and variety 3 (V3) at 7th, 14th, 21st and 28th DAI as challenged by *M. oryzae*. T0 = Absolute control, T1 = Negative control, T2 = Positive control, T3 = Seed priming, T4 = Seedling priming, T5 = Bacterial culture filtrate (BCF) foliar application, T6 = Seed priming + BCF foliar application and T7 = Seedling priming + BCF foliar application.

https://doi.org/10.1371/journal.pone.0351650.g007

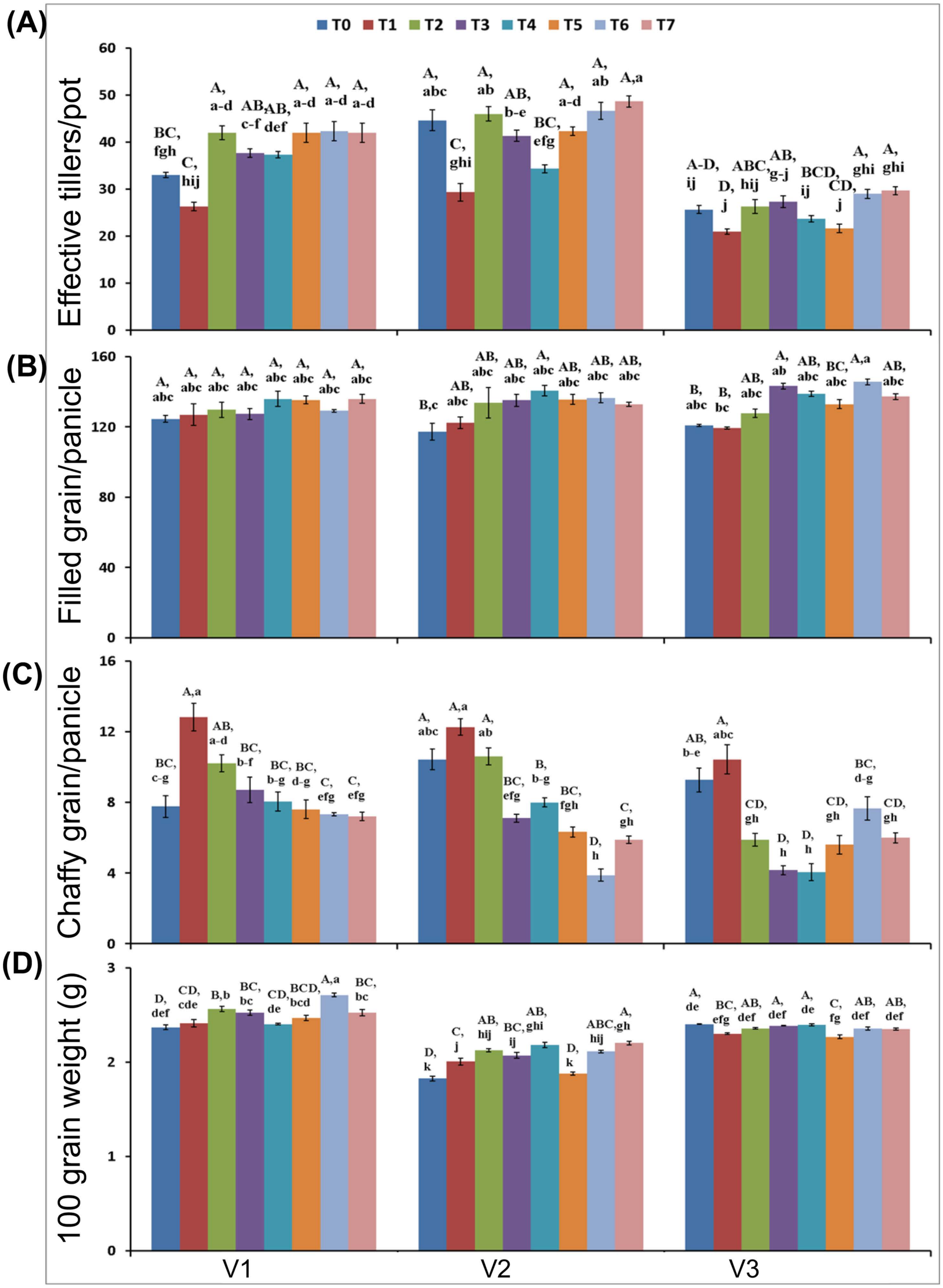

Fig 8. Effect of application of PR5 on yield traits of rice in variety I (V1), variety 2 (V2) and verity 3 (V3) as challenged by *M. oryzae*. (A) Effective tillers, **(B)** Filled grain, **(C)** Chaffy grain, **(D)** 100 grain weight. Capital letters represent the significant variations among the treatments within a variety

and small letters represent the variations among the treatments under three varieties (Tukey's test, $P < 0.05$). Bar represents the mean ± standard error (n = 3). T0 = Absolute control, T1 = Negative control, T2 = Positive control, T3 = Seed priming, T4 = Seedling priming, T5 = Bacterial culture filtrate (BCF) foliar application, T6 = Seed priming + BCF foliar application and T7 = Seedling priming + BCF foliar application.

https://doi.org/10.1371/journal.pone.0351650.g008

SeP + BCF produced the greatest yield, which was statistically similar to SP + BCF (Fig 9C). Notably, bacterial inoculation in SP + BCF and SeP + BCF treatments outperformed the chemical fungicide in enhancing both yield and yield attributes.

4 Discussion

Application of biological agents for controlling different plant diseases instead of chemical pesticides has gained research attention in recent years due to the environmental and ecosystem benefits offered by the approach. In this regard, *Pseudomonas* has shown great potential in controlling rice blast disease [54–57]. *P. mosselii* has been reported to have biocontrol potential against rice blast in several earlier reports [36,37], while *P. mosselii* PR5 has been reported to control naturally occurring rice blast [48]. In the current study, when the blast pathogen was applied in all the treatments except the absolute control, the bacterium showed great potential in controlling rice blast disease, comparable to the chemical fungicide and sometimes even more than the chemical fungicide in some treatments. Especially when culture filtrate of PR5 was applied in addition to seed priming or seedling priming, it outperformed the chemical fungicide not only in disease control but also in several other growth and yield traits.

In the case of the growth response of rice plants, a remarkable improvement in growth was observed in shoot growth in bacterium-treated plants at the early stage. The effect continued when plants were challenged by the blast pathogen at the tillering stage. The result was revealed at all the parameters at harvest with an increased root and shoot biomass production. The dry and fresh shoot and root weight was remarkably higher in plants primed with bacteria than in the plants that received only the pathogen, and even more than in the absolute control that did not receive the blast pathogen. The results proved that PR5 boosted plant growth under no stress or under biotic stress conferred by *M. oryzae.* The mechanism of growth improvement by PR5 was reported in earlier studies, where this bacterium was found capable of producing significant amounts of indole-3 acetic acid (IAA), fixing atmospheric nitrogen, solubilize insoluble phosphorus, Fe, zinc, and silicon [47,48]. IAA is responsible for root and shoot elongation along with lateral and fibrous root formation that significantly alters the root architecture [58,59]. The improved root growth enabled the plants to forage for more water and nutrients to support higher plant growth. Additionally, the availability of nutrients at an increased level in the soil enables plants to take up more nutrients, which also supports the plants' healthy growth and improves the yield attributes.

Upon application with the blast pathogen, the disease severity was reduced by the bacterial application by dual pathway. The first pathway of controlling the blast disease is the direct inhibition of the pathogenic strain by the bacterium, mainly through the production of antifungal compounds to inhibit the mycelial growth or conidial formations, as in the case of chemical fungicide. *P. mosselii* 923 has been reported to produce a natural pyrazolotriazine named pseudoiodinine to protect against the rice blast pathogen [36], while *P. putida* BP25 has been reported to produce antifungal pyrazines, which are responsible for defence against rice blast [34]. Pseudopyronine is another antimicrobial molecule which has been identified from *P. mosselii* [60]. Several earlier studies also reported some responsible genes for the disease suppression. *The gene cluster c-xtl is* reported to be responsible for blast disease control in *P. mosselii* BS011 [37]. *P. putida* stimulated defence against rice blast disease through cell wall polysaccharides [32]. Additionally, PR5 is capable of producing siderophore and HCN that protect the pathogenic fungi in soil. These two biomolecules helped to induce systemic resistance (ISR) in plants against plant pathogens and hence control the severity of *M. oryzae* as reported by Suresh et al., 2022 [61] for *Pseudomonas fluorescens* VSMKU3054 in tomato against *Ralstonia solanacearum*. According to de Vleesschauwer [38], pseudobactin-type siderophore produced by *P. fluorescens* WCS374r is a key determining factor for ISR in rice against *M. oryzae.* The ability of an organism to produce siderophore is closely related to cyanide production,

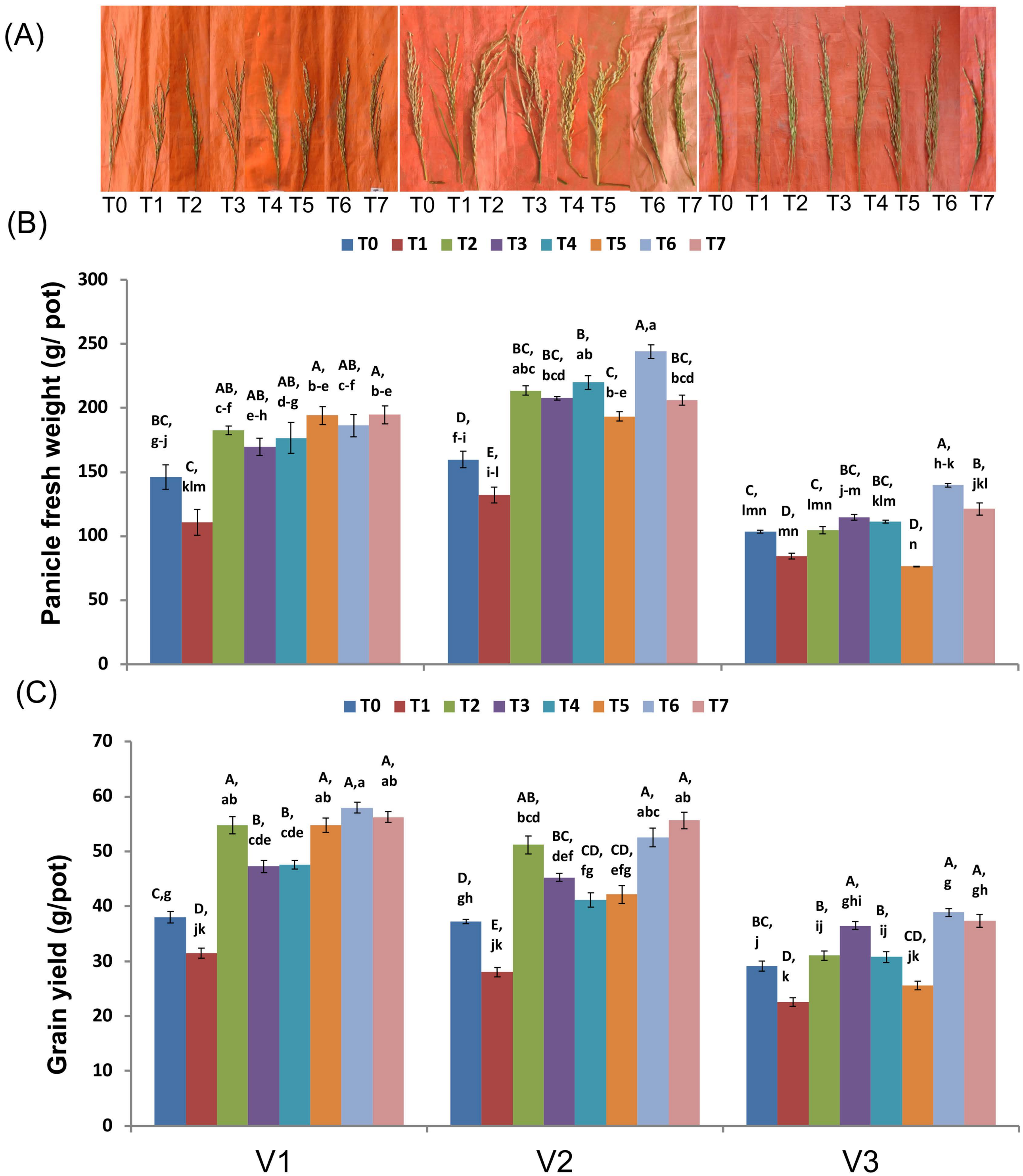

Fig 9. Effect of application of PR5 on yield traits of rice in variety I (V1), variety 2 (V2) and verity 3 (V3) as challenged by *M. oryzae*. (A) Visual observation of panicles at harvest, **(B)** Panicle fresh weight, **(C)** Grain yield. Capital letters represent the significant variations among the treatments within a variety and small letters represent the variations among the treatments under three varieties (Tukey's test, $P<0.05$). Bar represents the mean with standard error (n = 3). T0 = Absolute control, T1 = Negative control, T2 = Positive control, T3 = Seed priming, T4 = Seedling priming, T5 = Bacterial culture filtrate (BCF) foliar application, T6 = Seed priming + BCF foliar application and T7 = Seedling priming + BCF foliar application.

https://doi.org/10.1371/journal.pone.0351650.g009

and their absence can impact the biocontrol activity of the microbes and their ability to restrict iron access to target pathogens [62,63]. As already mentioned, the strain PR5 is able to produce both siderophore and HCN that might act as a crucial mechanism for elicitation of ISR in rice against pathogens in the current study. Iron plays an important role in the life of all living organisms [64]. In a natural environment at neutral pH, the dominant form of iron is in its oxidized ferric (Fe^{3+}) form. Siderophores act as high-affinity chelating agents to solubilize ferric ion and transport it to the cell where it converts to Fe2+ [65]; hence, limiting the supply of iron to the target pathogen, which can be used as a strategy of PR5 to restrict or inhibit their growth in the current experiment.

The second pathway of disease control is the growth promotion and nutrient absorption by the application of bacterium [66,67]. It is already mentioned that PR5 increased root surface and root biomass through altering root architecture, as supported by several earlier research studies [48]. The healthy plants with better root systems can protect against pathogenic infection significantly better than that of chemical fungicide-treated plants, as described in several earlier reports [68,69]. This is the significant difference between the chemical fungicide and the PR5 as a dual-resource biocontrol agent, where the chemical fungicide only protects the blast infection by direct mechanism, while the PR5 improves the growth and protects the blast pathogen pathogenic infection through a dual pathway. More importantly, the growth parameters and yield attributes of rice treated with PR5 were even better than the absolute control, indicating the superior plant growth promotion and pathogenic suppression potentiality of the bacterium even under M. oryzae infestation. Notably, plants in the absolute control treatment also showed blast infection, though plants subjected to MO challenges were physically separated by polythene sheets in a close chamber during disease inoculation. The reason might be the maintenance of near-natural field conditions with a 25 cm distance among the pots throughout the growing period except during disease inoculation. In addition, the plants were grown in the open experimental site where natural blast infestation could occur due to air borne pathogens spread in the environment.

Overall, the application of PR5 as a dual-resource biocontrol agent has shown great potentiality in controlling artificially inoculated rice blast and improved the plant growth and yield under *M. oryzae*-challenged conditions. The bacterium performed best when applied through seed or seedling priming along with foliar application. This might be because PR5 induced systemic resistance in the plants when primed in the seed or seedling and exhibited maximum growth-promoting traits as an endophyte. In the later stage, when additional foliar spray was done after pathogenic infection, it protected the pathogen through direct antagonism. Therefore, it could suggest that seed priming or seedling priming along with foliar applications could be the best strategy to control rice blast and to promote rice growth and yield without using chemical fungicide.

5 Conclusion

The PGPR inoculum, *Pseudomonas mosselii* PR5 showed strong potential as a dual-function biocontrol agent by effectively controlling rice blast disease and improving plant growth and grain yield across susceptible rice genotypes. The better performance of PR5 applications over chemical fungicides under both control and pathogen-challenged conditions is likely related to its several beneficial mechanisms, including direct antagonism against the pathogen, induction of systemic resistance, and stimulation of root growth and nutrient uptake mechanisms. The research findings also suggest that combining seed or seedling priming with preventive and protective foliar sprays can provide a more effective management strategy for rice blast disease control while promoting crop productivity. Beyond the role of PR5 in controlling diseases, this research demonstrates that PR5 has important agricultural benefits for sustainable agricultural practices. The single microbial agent provides the practical solution which reduces synthetic fungicide usage while protecting the environment through its dual benefits of plant health improvement and disease resistance and yield enhancement. The integration of PR5 into rice production system will help farmers achieve environmentally friendly blast disease management practices to boost productivity and lower chemical inputs in rice farming.

Author contributions

Conceptualization: Razia Sultana, Shah Mohammad Naimul Islam.

Data curation: Razia Sultana, Prinon Saha, Sourav Biswas Shuvo, Md. Mustafijur Rahman Khan, Seikh Jafor Ahmed.

Formal analysis: Prinon Saha, Sourav Biswas Shuvo, Md. Mustafijur Rahman Khan, Seikh Jafor Ahmed.

Funding acquisition: Razia Sultana.

Investigation: Razia Sultana, Shah Mohammad Naimul Islam, Mohammad Mahbubul Haque.

Methodology: Razia Sultana.

Project administration: Razia Sultana, Mohammad Mahbubul Haque.

Resources: Razia Sultana, Shah Mohammad Naimul Islam, Mohammad Mahbubul Haque.

Software: Prinon Saha.

Supervision: Razia Sultana.

Validation: Razia Sultana, Prinon Saha, Shah Mohammad Naimul Islam.

Visualization: Razia Sultana, Shah Mohammad Naimul Islam.

Writing – original draft: Razia Sultana.

Writing – review & editing: Razia Sultana, Prinon Saha, Shah Mohammad Naimul Islam, Mohammad Mahbubul Haque, Sourav Biswas Shuvo, Md. Mustafijur Rahman Khan, Seikh Jafor Ahmed.

References

1. Mohidem NA, Hashim N, Shamsudin R, Che Man H. Rice for Food Security: Revisiting Its Production, Diversity, Rice Milling Process and Nutrient Content. Agriculture. 2022;12(6):741. https://doi.org/10.3390/agriculture12060741
2. Shi J, An G, Weber APM, Zhang D. Prospects for rice in 2050. Plant Cell Environ. 2023;46(4):1037–45. https://doi.org/10.1111/pce.14565 PMID: 36805595
3. Kumar U, Kaviraj M, Kundu S, Rout S, Priya H, Nayak AK. Microbial Alleviation of Abiotic and Biotic Stresses in Rice. Sustainable Agriculture Reviews. Springer Nature Switzerland. 2023. 243–68. https://doi.org/10.1007/978-3-031-24181-9_10
4. Sakulkoo W, Osés-Ruiz M, Oliveira Garcia E, Soanes DM, Littlejohn GR, Hacker C, et al. A single fungal MAP kinase controls plant cell-to-cell invasion by the rice blast fungus. Science. 2018;359(6382):1399–403. https://doi.org/10.1126/science.aaq0892 PMID: 29567712
5. Devanna BN, Jain P, Solanke AU, Das A, Thakur S, Singh PK, et al. Understanding the Dynamics of Blast Resistance in Rice-Magnaporthe oryzae Interactions. J Fungi (Basel). 2022;8(6):584. https://doi.org/10.3390/jof8060584 PMID: 35736067
6. Seebold KW Jr, Datnoff LE, Correa-Victoria FJ, Kucharek TA, Snyder GH. Effects of Silicon and Fungicides on the Control of Leaf and Neck Blast in Upland Rice. Plant Dis. 2004;88(3):253–8. https://doi.org/10.1094/PDIS.2004.88.3.253 PMID: 30812356
7. Chen X, Jia Y, Wu BM. Evaluation of Rice Responses to the Blast Fungus Magnaporthe oryzae at Different Growth Stages. Plant Dis. 2019;103(1):132–6. https://doi.org/10.1094/PDIS-12-17-1873-RE PMID: 30444467
8. Kumar M, Ashraf S. Integrated Management of Rice Blast Caused by Magnaporthe oryzae. Plant Health Under Biotic Stress. Springer Singapore. 2019. 91–105. https://doi.org/10.1007/978-981-13-6043-5_5
9. Pahalvi HN, Rafiya L, Rashid S, Nisar B, Kamili AN. Chemical Fertilizers and Their Impact on Soil Health. Microbiota and Biofertilizers, Vol 2. Springer International Publishing. 2021. 1–20. https://doi.org/10.1007/978-3-030-61010-4_1
10. Corkley I, Fraaije B, Hawkins N. Fungicide resistance management: Maximizing the effective life of plant protection products. Plant Pathology. 2021;71(1):150–69. https://doi.org/10.1111/ppa.13467
11. Ahmad MF, Ahmad FA, Alsayegh AA, Zeyaullah M, AlShahrani AM, Muzammil K, et al. Pesticides impacts on human health and the environment with their mechanisms of action and possible countermeasures. Heliyon. 2024;10(7):e29128. https://doi.org/10.1016/j.heliyon.2024.e29128 PMID: 38623208
12. Saqib S, Zaman W, Ullah F, Majeed I, Ayaz A, Hussain Munis MF. Organometallic assembling of chitosan-iron oxide nanoparticles with their antifungal evaluation against Rhizopus oryzae. Applied Organometallic Chemistry. 2019;33(11):e5190. https://doi.org/10.1002/aoc.5190
13. Saqib S, Zaman W, Ayaz A, Habib S, Bahadur S, Hussain S, et al. Postharvest disease inhibition in fruit by synthesis and characterization of chitosan iron oxide nanoparticles. Biocatalysis and Agricultural Biotechnology. 2020;28:101729. https://doi.org/10.1016/j.bcab.2020.101729

14. Beyari EA. Alternatives to chemical pesticides: the role of microbial biocontrol agents in phytopathogen management: a comprehensive review. J Plant Pathol. 2024;107(1):291–314. https://doi.org/10.1007/s42161-024-01808-8
15. Koul B, Chopra M, Lamba S. Microorganisms as biocontrol agents for sustainable agriculture. Relationship Between Microbes and the Environment for Sustainable Ecosystem Services, Volume 1. Elsevier. 2022. 45–68. https://doi.org/10.1016/b978-0-323-89938-3.00003-7
16. Palmieri D, Ianiri G, Del Grosso C, Barone G, De Curtis F, Castoria R, et al. Advances and Perspectives in the Use of Biocontrol Agents against Fungal Plant Diseases. Horticulturae. 2022;8(7):577. https://doi.org/10.3390/horticulturae8070577
17. Alqahtani FS. The utilization of microorganisms for biological control of soil-borne plant pathogens: a sustainable strategy for managing plant diseases - a comprehensive review. J Plant Pathol. 2025;107(4):1815–39. https://doi.org/10.1007/s42161-025-01984-1
18. Kumar S, Nikunj C, Rathore A, Nigam R, Shivam, Mohanan A, et al. Role of Biocontrol Agents in Suppressing Plant Pathogen: A Compressive Analysis. J Sci Res Rep. 2024;30(10):1004–15. https://doi.org/10.9734/jsrr/2024/v30i102522
19. Villavicencio-Vásquez M, Espinoza-Lozano F, Espinoza-Lozano L, Coronel-León J. Biological control agents: mechanisms of action, selection, formulation and challenges in agriculture. Front Agron. 2025;7. https://doi.org/10.3389/fagro.2025.1578915
20. Sheoran AR, Lakra N, Saharan BS, Luhach A, Kumar R, Seth CS, et al. Enhancing Plant Disease Resistance: Insights from Biocontrol Agent Strategies. J Plant Growth Regul. 2024;44(2):436–59. https://doi.org/10.1007/s00344-024-11480-y
21. Sunera, Khan Z, Irshad M, Zakria M, Saqib S, Zaman W. Evaluating the efficacy of endophytic bacteria in controlling rice sheath blight: In vitro and In vivo studies. Microb Pathog. 2024;197:107084. https://doi.org/10.1016/j.micpath.2024.107084 PMID: 39490941
22. Gowtham HG, Hariprasad P, Nayak SC, Niranjana SR. Application of rhizobacteria antagonistic to Fusarium oxysporum f. sp. lycopersici for the management of Fusarium wilt in tomato. Rhizosphere. 2016;2:72–4. https://doi.org/10.1016/j.rhisph.2016.07.008
23. Sahu KP, Kumar A, Patel A, Kumar M, Gopalakrishnan S, Prakash G, et al. Rice Blast Lesions: an Unexplored Phyllosphere Microhabitat for Novel Antagonistic Bacterial Species Against Magnaporthe oryzae. Microb Ecol. 2021;81(3):731–45. https://doi.org/10.1007/s00248-020-01617-3 PMID: 33108474
24. Zhou H, Ren Z-H, Zu X, Yu X-Y, Zhu H-J, Li X-J, et al. Efficacy of Plant Growth-Promoting Bacteria Bacillus cereus YN917 for Biocontrol of Rice Blast. Front Microbiol. 2021;12:684888. https://doi.org/10.3389/fmicb.2021.684888 PMID: 34354684
25. El-Saadony MT, Saad AM, Soliman SM, Salem HM, Ahmed AI, Mahmood M, et al. Plant growth-promoting microorganisms as biocontrol agents of plant diseases: Mechanisms, challenges and future perspectives. Front Plant Sci. 2022;13:923880. https://doi.org/10.3389/fpls.2022.923880 PMID: 36275556
26. Islam SMN, Chowdhury MZH, Mim MF, Momtaz MB, Islam T. Biocontrol potential of native isolates of Beauveria bassiana against cotton leafworm Spodoptera litura (Fabricius). Sci Rep. 2023;13(1):8331. https://doi.org/10.1038/s41598-023-35415-x PMID: 37221248
27. Balthazar C, Novinscak A, Cantin G, Joly DL, Filion M. Biocontrol Activity of Bacillus spp. and Pseudomonas spp. Against Botrytis cinerea and Other Cannabis Fungal Pathogens. Phytopathology. 2022;112(3):549–60. https://doi.org/10.1094/PHYTO-03-21-0128-R PMID: 34293909
28. Mehmood N, Saeed M, Zafarullah S, Hyder S, Rizvi ZF, Gondal AS, et al. Multifaceted Impacts of Plant-Beneficial Pseudomonas spp. in Managing Various Plant Diseases and Crop Yield Improvement. ACS Omega. 2023;8(25):22296–315. https://doi.org/10.1021/acsomega.3c00870 PMID: 37396244
29. Li C, Gao X, Huo Y, Asseri TAY, Tian X, Luo K. Evaluation of biocontrol efficacy of rhizosphere Pseudomonas aeruginosa for management of Phytophthora capsici of pepper. PLoS One. 2024;19(9):e0309705. https://doi.org/10.1371/journal.pone.0309705 PMID: 39302985
30. Ghadamgahi F, Tarighi S, Taheri P, Saripella GV, Anzalone A, Kalyandurg PB, et al. Plant Growth-Promoting Activity of Pseudomonas aeruginosa FG106 and Its Ability to Act as a Biocontrol Agent against Potato, Tomato and Taro Pathogens. Biology (Basel). 2022;11(1):140. https://doi.org/10.3390/biology11010140 PMID: 35053136
31. Arseneault T, Filion M. Phenazine-Producing Pseudomonas spp. as Biocontrol Agents of Plant Pathogens. In: Singh D, Singh H, Prabha R. Microbial Inoculants in Sustainable Agricultural Productivity. New Delhi: Springer. 2016. https://doi.org/10.1007/978-81-322-2644-4
32. Ashajyothi M, Balamurugan A, Patel A, Krishnappa C, Kumar R, Kumar A. Cell wall polysaccharides of endophytic Pseudomonas putida elicit defense against rice blast disease. J Appl Microbiol. 2023;134(2):lxac042. https://doi.org/10.1093/jambio/lxac042 PMID: 36626745
33. Abo-Elyousr KAM, Abdel-Rahim IR, Almasoudi NM, Alghamdi SA. Native Endophytic Pseudomonas putida as a Biocontrol Agent against Common Bean Rust Caused by Uromyces appendiculatus. J Fungi (Basel). 2021;7(9):745. https://doi.org/10.3390/jof7090745 PMID: 34575783
34. Patel A, Kumar A, Sheoran N, Kumar M, Sahu KP, Ganeshan P, et al. Antifungal and defense elicitor activities of pyrazines identified in endophytic Pseudomonas putida BP25 against fungal blast incited by Magnaporthe oryzae in rice. J Plant Dis Prot. 2020;128(1):261–72. https://doi.org/10.1007/s41348-020-00373-3
35. Widiantini F, Herdiansyah A, Yulia E. Biocontrol Potential of Endophytic Bacteria Isolated from Healthy Rice Plant against Rice Blast Disease (Pyricularia oryzae Cav.). KLS. 2017;2(6):287. https://doi.org/10.18502/kls.v2i6.1051
36. Yang R, Shi Q, Huang T, Yan Y, Li S, Fang Y, et al. The natural pyrazolotriazine pseudoiodinine from Pseudomonas mosselii 923 inhibits plant bacterial and fungal pathogens. Nat Commun. 2023;14(1):734. https://doi.org/10.1038/s41467-023-36433-z PMID: 36759518
37. Wu L, Xiao W, Chen G, Song D, Khaskheli MA, Li P, et al. Identification of Pseudomonas mosselii BS011 gene clusters required for suppression of Rice Blast Fungus Magnaporthe oryzae. J Biotechnol. 2018;282:1–9. https://doi.org/10.1016/j.jbiotec.2018.04.016 PMID: 29704539

38. De Vleesschauwer D, Djavaheri M, Bakker PAHM, Höfte M. Pseudomonas fluorescens WCS374r-induced systemic resistance in rice against Magnaporthe oryzae is based on pseudobactin-mediated priming for a salicylic acid-repressible multifaceted defense response. Plant Physiol. 2008;148(4):1996–2012. https://doi.org/10.1104/pp.108.127878 PMID: 18945932

39. Mitra D, Mondal R, Khoshru B, Shadangi S, Das Mohapatra PK, Panneerselvam P. Rhizobacteria mediated seed bio-priming triggers the resistance and plant growth for sustainable crop production. Curr Res Microb Sci. 2021;2:100071. https://doi.org/10.1016/j.crmicr.2021.100071 PMID: 34841361

40. Fiodor A, Ajijah N, Dziewit L, Pranaw K. Biopriming of seed with plant growth-promoting bacteria for improved germination and seedling growth. Front Microbiol. 2023;14:1142966. https://doi.org/10.3389/fmicb.2023.1142966 PMID: 36925481

41. Sultana R, Islam SMN, Shuvo SB, Ehsan GMdA, Saha P, Khan MdMR, et al. Endophytic bacterium Sphingomonas panaciterrae NB5 influences soil properties and improves growth, nutrient contents, and yield of red amaranth (Amaranthus tricolor L.). Current Plant Biology. 2024;39:100372. https://doi.org/10.1016/j.cpb.2024.100372

42. Fukami J, Ollero FJ, Megías M, Hungria M. Phytohormones and induction of plant-stress tolerance and defense genes by seed and foliar inoculation with Azospirillum brasilense cells and metabolites promote maize growth. AMB Express. 2017;7(1):153. https://doi.org/10.1186/s13568-017-0453-7 PMID: 28724262

43. Efthimiadou A, Katsenios N, Chanioti S, Giannoglou M, Djordjevic N, Katsaros G. Effect of foliar and soil application of plant growth promoting bacteria on growth, physiology, yield and seed quality of maize under Mediterranean conditions. Sci Rep. 2020;10(1):21060. https://doi.org/10.1038/s41598-020-78034-6 PMID: 33273634

44. Freitas GS, Moreira A, Prudencio MF, Heinrichs R, Nogueira MA, Hungria M, et al. Foliar Spray Inoculation with Plant Growth Promoting Bacteria Associated with Nitrogen Doses in Megathyrsus maximus cv. BRS Zuri. Agronomy. 2023;13(4):1040. https://doi.org/10.3390/agronomy13041040

45. Sultana R, Islam SMN, Sriti N, Ahmed M, Shuvo SB, Rahman MH, et al. Sphingomonas panaciterrae PB20 increases growth, photosynthetic pigments, antioxidants, and mineral nutrient contents in spinach (Spinacia oleracea L.). Heliyon. 2024;10(3):e25596. https://doi.org/10.1016/j.heliyon.2024.e25596 PMID: 38356594

46. Duarte B, Carreiras JA, Cruz-Silva A, Mateos-Naranjo E, Rodríguez-Llorente ID, Pajuelo E, et al. Marine Plant Growth Promoting Bacteria (PGPB) inoculation technology: Testing the effectiveness of different application methods to improve tomato plants tolerance against acute heat wave stress. Plant Stress. 2024;11:100434. https://doi.org/10.1016/j.stress.2024.100434

47. Sultana R, Islam SMN, Sultana T. Arsenic and other heavy metals resistant bacteria in rice ecosystem: Potential role in promoting plant growth and tolerance to heavy metal stress. Environmental Technology & Innovation. 2023;31:103160. https://doi.org/10.1016/j.eti.2023.103160

48. Sultana R, Jashim AII, Islam SMN, Rahman MH, Haque MM. Bacterial endophyte *Pseudomonas mosselii* PR5 improves growth, nutrient accumulation, and yield of rice (*Oryza sativa* L.) through various application methods. BMC Plant Biology. 2024c; 24:1030. DOI:10.1186/s12870-024-05649-6

49. Ahmmed S, Jahiruddin M, Razia S, Begum RA, Biswas JC, Rahman ASMM, et al. Fertilizer Recommendation Guide-2018. Dhaka, Bangladesh: Bangladesh Agricultural Research Council (BARC). 2018.

50. Rafi A, Hameed A, Akhtar MA, Shah SM, Junaid M, Shahid M, Shah SF. Field based assessment of rice bacterial leaf blight in major rice growing zones of Pakistan. Sarhad Journal of Agriculture. 2013;29(3):415–22.

51. Maclean J, Hardy B, Hettel G. Rice Almanac: Source book for one of the most important economic activities on earth. Los Baños, Philippines: International Rice Research Institute (IRRI). 2013.

52. Baker KF, Wheeler BEJ. An Introduction to Plant Diseases. Mycologia. 1970;62(3):617. https://doi.org/10.2307/3757541

53. Shaner G, Finney RE. The Effect of Nitrogen Fertilization on the Expression of Slow-Mildewing Resistance in Knox Wheat. Phytopathology. 2025;115(8V):1051–6. https://doi.org/10.1094/Phyto-67-1051 PMID: 40934422

54. Jambhulkar PP, Sharma P, Manokaran R, Lakshman DK, Rokadia P, Jambhulkar N. Assessing synergism of combined applications of Trichoderma harzianum and Pseudomonas fluorescens to control blast and bacterial leaf blight of rice. Eur J Plant Pathol. 2018;152(3):747–57. https://doi.org/10.1007/s10658-018-1519-3

55. Omoboye OO, Oni FE, Batool H, Yimer HZ, De Mot R, Höfte M. Pseudomonas Cyclic Lipopeptides Suppress the Rice Blast Fungus Magnaporthe oryzae by Induced Resistance and Direct Antagonism. Front Plant Sci. 2019;10:901. https://doi.org/10.3389/fpls.2019.00901 PMID: 31354771

56. Jayamohan NS, Patil SV, Kumudini BS. Seed priming with Pseudomonas putida isolated from rhizosphere triggers innate resistance against Fusarium wilt in tomato through pathogenesis-related protein activation and phenylpropanoid pathway. Pedosphere. 2020;30(5):651–60. https://doi.org/10.1016/S1002-0160(20)60027-3

57. Zeng Q, Man X, Huang Z, Zhuang L, Yang H, Sha Y. Effects of rice blast biocontrol strain Pseudomonas alcaliphila Ej2 on the endophytic microbiome and proteome of rice under salt stress. Front Microbiol. 2023;14:1129614. https://doi.org/10.3389/fmicb.2023.1129614 PMID: 36960288

58. Vacheron J, Desbrosses G, Bouffaud M-L, Touraine B, Moënne-Loccoz Y, Muller D, et al. Plant growth-promoting rhizobacteria and root system functioning. Front Plant Sci. 2013;4:356. https://doi.org/10.3389/fpls.2013.00356 PMID: 24062756

59. Singh P, Singh RK, Zhou Y, Wang J, Jiang Y, Shen N, et al. Unlocking the strength of plant growth promoting Pseudomonas in improving crop productivity in normal and challenging environments: a review. Journal of Plant Interactions. 2022;17(1):220–38. https://doi.org/10.1080/17429145.2022.2029963

60. Nishanth Kumar S, Aravind SR, Jacob J, Gopinath G, Lankalapalli RS, Sreelekha TT, et al. Pseudopyronine B: a potent antimicrobial and anticancer molecule isolated from a Pseudomonas mosselii. Front Microbiol. 2016;7:1307. https://doi.org/10.3389/fmicb.2016.01307

61. Suresh P, Shanmugaiah V, Rajagopal R, Muthusamy K, Ramamoorthy V. Pseudomonas fluorescens VSMKU3054 mediated induced systemic resistance in tomato against Ralstonia solanacearum. Physiological and Molecular Plant Pathology. 2022;119:101836. https://doi.org/10.1016/j.pmpp.2022.101836

62. Jha CK, Aeron A, Patel BV, Maheshwari DK, Saraf M. Enterobacter: role in plant growth promotion. Bacteria in Agrobiology: Plant Growth Responses. Berlin, Heidelberg: Springer Berlin Heidelberg. 2011. 159–82. https://doi.org/10.1007/978-3-642-20332-9_8

63. Ho Y-N, Hoo SY, Wang B-W, Hsieh C-T, Lin C-C, Sun C-H, et al. Specific inactivation of an antifungal bacterial siderophore by a fungal plant pathogen. ISME J. 2021;15(6):1858–61. https://doi.org/10.1038/s41396-020-00871-0 PMID: 33619352

64. Haschka D, Hoffmann A, Weiss G. Iron in immune cell function and host defense. Semin Cell Dev Biol. 2021;115:27–36. https://doi.org/10.1016/j.semcdb.2020.12.005 PMID: 33386235

65. Qing-Ping Hu. A simple double-layered chrome azurol S agar (SD-CASA) plate assay to optimize the production of siderophores by a potential biocontrol agent Bacillus. Afr J Microbiol Res. 2011;5(25). https://doi.org/10.5897/ajmr11.238

66. Salomon MV, Funes Pinter I, Piccoli P, Bottini R. Use of Plant Growth-Promoting Rhizobacteria as Biocontrol Agents: Induced Systemic Resistance Against Biotic Stress in Plants. Microbial Applications Vol.2. Springer International Publishing. 2017. 133–52. https://doi.org/10.1007/978-3-319-52669-0_7

67. Abd El-Mageed TA, Rady MM, Taha RS, Abd El Azeam S, Simpson CR, Semida WM. Effects of integrated use of residual sulfur-enhanced biochar with effective microorganisms on soil properties, plant growth and short-term productivity of Capsicum annuum under salt stress. Scientia Horticulturae. 2020;261:108930. https://doi.org/10.1016/j.scienta.2019.108930

68. Bardin M, Ajouz S, Comby M, Lopez-Ferber M, Graillot B, Siegwart M, et al. Is the efficacy of biological control against plant diseases likely to be more durable than that of chemical pesticides?. Front Plant Sci. 2015;6:566. https://doi.org/10.3389/fpls.2015.00566 PMID: 26284088

69. Deresa EM, Diriba TF. Phytochemicals as alternative fungicides for controlling plant diseases: A comprehensive review of their efficacy, commercial representatives, advantages, challenges for adoption, and possible solutions. Heliyon. 2023;9(3):e13810. https://doi.org/10.1016/j.heliyon.2023.e13810 PMID: 36879959
